# Activation of Focal Adhesion Kinase by *Salmonella* Suppresses Autophagy via an Akt/mTOR Signaling Pathway and Promotes Bacterial Survival in Macrophages

**DOI:** 10.1371/journal.ppat.1004159

**Published:** 2014-06-05

**Authors:** Katherine A. Owen, Corey B. Meyer, Amy H. Bouton, James E. Casanova

**Affiliations:** 1 Department of Cell Biology, University of Virginia Health System, Charlottesville, Virginia, United States of America; 2 Department of Microbiology, University of Virginia Health System, Charlottesville, Virginia, United States of America; University of New Mexico, United States of America

## Abstract

Autophagy has emerged as an important antimicrobial host defense mechanism that not only orchestrates the systemic immune response, but also functions in a cell autonomous manner to directly eliminate invading pathogens. Pathogenic bacteria such as *Salmonella* have evolved adaptations to protect themselves from autophagic elimination. Here we show that signaling through the non-receptor tyrosine kinase focal adhesion kinase (FAK) is actively manipulated by the *Salmonella* SPI-2 system in macrophages to promote intracellular survival. In wild-type macrophages, FAK is recruited to the surface of the *Salmonella*-containing vacuole (SCV), leading to amplified signaling through the Akt-mTOR axis and inhibition of the autophagic response. In FAK-deficient macrophages, Akt/mTOR signaling is attenuated and autophagic capture of intracellular bacteria is enhanced, resulting in reduced bacterial survival. We further demonstrate that enhanced autophagy in FAK^−/−^ macrophages requires the activity of Atg5 and ULK1 in a process that is distinct from LC3-assisted phagocytosis (LAP). *In vivo*, selective knockout of FAK in macrophages resulted in more rapid clearance of bacteria from tissues after oral infection with *S. typhimurium*. Clearance was correlated with reduced infiltration of inflammatory cell types into infected tissues and reduced tissue damage. Together, these data demonstrate that FAK is specifically targeted by *S. typhimurium* as a novel means of suppressing autophagy in macrophages, thereby enhancing their intracellular survival.

## Author Summary

*Salmonella enterica* is a food- and water-borne pathogen that has evolved closely with vertebrate hosts. Two medically relevant serovars include *S. typhimurium*, which causes gastroenteritis and *S. typhi*, which is the causative agent of typhoid fever. Host cells can utilize a process called autophagy, normally involved in the elimination of defective proteins and organelles, to capture and degrade intracellular pathogens. Enteric *Salmonella* express numerous virulence factors that enable the bacterium to subvert host defense mechanisms. Here we report that *Salmonella* specifically activates the host molecule focal adhesion kinase (FAK) in macrophages, triggering a signaling cascade that suppresses the autophagic elimination of intracellular bacteria. A key regulator of autophagy in mammalian cells is the target of rapamycin, mTOR, which transmits inhibitory signals that downregulate the autophagic response. We show that *Salmonella*-induced FAK activation leads to the Akt-dependent activation of mTOR, thereby repressing autophagic signaling. Inhibition of autophagy results in increased bacterial survival, while in FAK-deficient cells, autophagy is enhanced and intracellular *Salmonella* are eliminated. We also show that in mice lacking macrophage-specific FAK, animals were less susceptible to oral *Salmonella* infection. Together, these data identify FAK as a novel regulator of autophagy in macrophages with broad implications for host survival.

## Introduction

Serovars of *Salmonella enterica* are facultative intracellular Gram-negative entero-pathogens that cause a spectrum of human diseases ranging from localized gastroenteritis to typhoid fever. Pathogenic *Salmonella* strains use two Type III secretion systems (T3SS), encoded within *Salmonella* Pathogenicity Islands I (SPI-1) and 2 (SPI-2) to translocate distinct arrays of virulence proteins into host cells. Although there is some functional overlap, effectors translocated via T3SS-1 facilitate bacterial penetration of the intestinal epithelium, while effectors translocated via T3SS-2 promote intracellular survival [1], [2]. Expression of T3SS-2 occurs 2–5 hours after entry of the bacteria into host cells and is necessary for remodeling of the phagosome into a specialized bacterial replication niche, the *Salmonella* containing vacuole (SCV) [2]. If local host defenses are insufficient to limit infection to the intestinal tract, bacteria disseminate systemically, first colonizing the mesenteric lymph nodes (mLNs), followed by the liver and spleen.

Autophagy is an evolutionarily conserved response to cellular stress whereby cytosolic components and/or organelles are sequestered inside double-membraned autophagosomes and delivered to lysosomes for degradation [3]. Although best known for its fundamental role in maintaining metabolic homeostasis, it is increasingly recognized that pathogenic bacteria interact with and are contained by autophagy systems [4], [5], [6]. Importantly, toll-like receptors (TLRs), which function as the major innate immune sensors for detecting specific molecular patterns expressed by pathogens, are linked to the induction of autophagy [6], [7], [8], [9]. In addition to canonical autophagy, a subset of autophagy components are involved in a related process, LC3-assisted phagocytosis (LAP), which does not lead to the formation of a double membrane-bound autophagosome [10], [11], [12], [13]. Nonetheless, LAP results in the degradation of cargo by promoting rapid phagosome-lysosome fusion [10]. This process requires the ubiquitin-like protein Atg5 but is independent of the canonical autophagy preinitiation complex consisting of ULK1/Atg13/FIP200 [14], [15], [16].

*Salmonella* is targeted by the autophagic machinery in epithelial cells, however recognition depends on expression of T3SS-1 [17], [18]. Active invasion of epithelial cells requires T3SS-1 and results in a transient permeabilization of the SCV [17], allowing components such as ubiquitin and the autophagic adaptor molecules p62, optineurin and NDP52 to target the damaged vacuoles [19], [20], [21], [22]. In contrast, the T3SS-2 is not believed to be involved in the autophagic targeting of SCV-bound *Salmonella* in non-phagocytic cells [23]. Unlike epithelial cells, macrophages are professional phagocytes that do not require direct invasion for efficient bacterial internalization. Macrophages play a crucial role in host defense through recognition and direct elimination of invading pathogens. Importantly, pathogens such as *Mycobacterium tuberculosis* and *Helicobacter pyloi* [24], [25], which are unable to escape phagosomal compartments, are sequestered within multimembrane compartments where they are degraded [24], [25]. While it is clear that autophagy is an integral part of the innate immune response to intracellular pathogens, how autophagosomes (or components of the autophagy system) capture pathogens that are contained within vacuolar compartments is not understood.

Focal adhesion kinase (FAK) is a non-receptor tyrosine kinase best known for its role in adhesion-mediated signaling in multiple cell types, including those of myeloid derivation [26]. In addition, FAK has been implicated in autophagy. A FAK-interacting protein, FIP200, is a component of the ULK1/ULK2 complex that acts at the earliest stages of autophagosome formation, and loss of FIP200 impairs autophagy [27]. Additionally, FAK can stimulate Akt activity [28], which functions as an important upstream regulator of the mammalian target of rapamycin complex 1 (mTORC1), a highly conserved serine/threonine kinase that acts as a sensor of growth factor/nutrient status and serves as a master regulator of autophagy. More recently, the loss of FAK itself was shown to enhance autophagic turnover in primary squamous carcinoma cells, although the pathway(s) leading from FAK to the autophagic response were not defined [29].

Here we show that the *Salmonella typhimurium* SPI-2 machinery actively manipulates FAK to suppress autophagic signaling in macrophages. We demonstrate that FAK is recruited to SCVs in a manner that is dependent upon the SPI-2 T3SS, where it promotes robust Akt activation and stimulation of the mTOR signaling pathway. FAK knockout prevents activation of the Akt-mTORC1 signaling axis by *S. typhimurium*, leading to more efficient capture of bacteria in autophagosomes and reduced intracellular survival. Furthermore, this autophagic targeting of bacteria requires the activity of both Atg5 and ULK1 and therefore appears to occur independently of LAP. Using a mouse model where FAK expression is selectively deleted from macrophages *in vivo*, we further demonstrate that FAK deficiency results in improved control of Salmonella infection and reduced inflammation in infected animals. Taken together, these results indicate that FAK is selectively targeted by Salmonella as a means of attenuating the host autophagic response, and highlight how the containment of intracellular pathogens contributes to host survival.

## Results

### FAK is recruited to SCVs in macrophages

**Figure 1:**
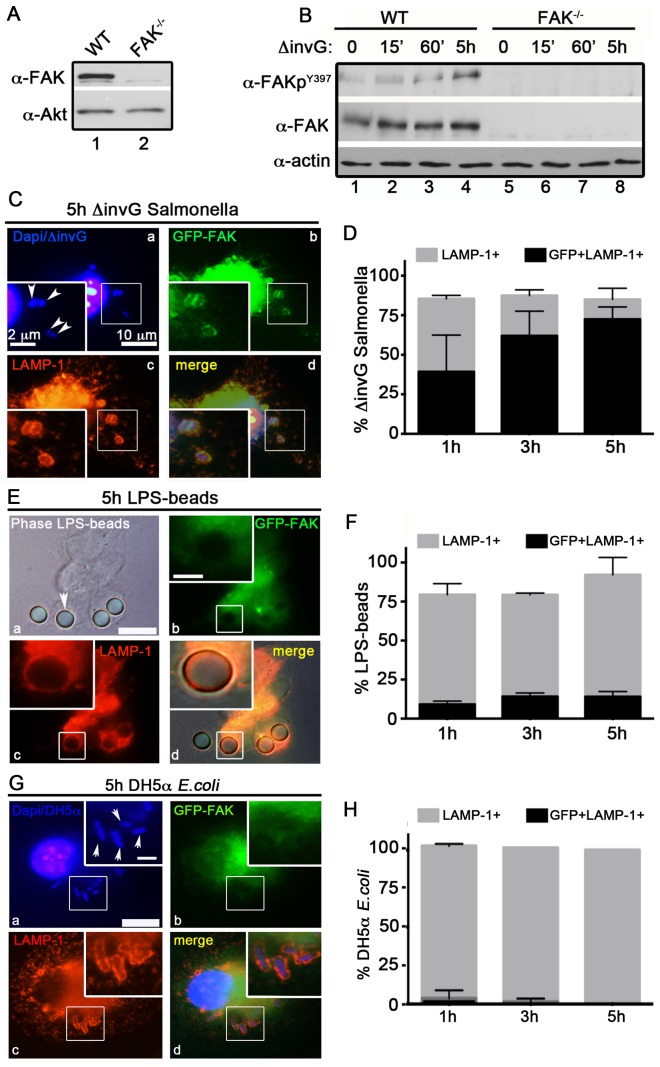
FAK is selectively recruited to LAMP-1-positive SCVs. (A) FAK immunoblotting was performed on macrophages isolated from the peritoneum of WT and FAK^Δmyeloid^ mice. (B) WT and FAK^−/−^ PEMs were incubated with *S. typhimurium* strain *ΔinvG* for 0–5 hours before immunoblotting with the indicated antibodies. (C, E and G) WT PEMs expressing GFP-FAK were incubated with either *ΔinvG Salmonella* (C), LPS-coated beads (E) or *E. coli* (G) for a total of 5 hours before analysis by immunofluorescence. Cells were co-stained with antibodies recognizing LAMP1 (red). Dapi was used to visualize nuclei and bacteria (blue). Bars represent 10 µm. Arrowheads indicate bacteria or beads in enlarged panel where bars represent 2 µm. (D, F and H) The percent of LAMP1-positive (gray bars) and LAMP1+GFP+ (black bars) *ΔinvG Salmonella* (D), LPS-coated beads (F) and *E. coli* (H) was quantified. At least 100 bacteria were counted per condition; N = 3.

Previously, we generated C57BL/6 mice lacking FAK in myeloid-lineage cells, using LysM-Cre [26]. The specificity of FAK deletion in primary peritoneal exudate macrophages (PEMs) was confirmed by immunoblot analysis (Figure 1A). We next wanted to determine how FAK functions in bacterial recognition signaling pathways in macrophages. Because invasive *Salmonella* rapidly kill macrophages through a SPI-1-dependent mechanism [30], we used a mutant of *S. typhimurium* strain SL1344 (*ΔinvG*) that lacks a functional SPI-1 T3SS. Since expression of invasion-associated genes is down-regulated after entry into host cells [2], we also reasoned that this strain would more closely mimic *Salmonella* as they are encountered by professional phagocytes after penetration of the epithelial barrier.

Stimulation of host macrophages with Gram-negative bacteria can trigger both NF-κB and MAPK signaling pathways [31]. As shown in Figure S1A and S1B, incubation of *ΔinvG Salmonella* with either WT or FAK^−/−^ PEMs resulted in the rapid and robust activation of ERK1/2, p38 and NF-κB, indicating that signaling through these pathways does not require FAK. In agreement with these observations, production of canonical NF-κB-dependent pro-inflammatory cytokines (TNF-α IL-6 and KC) was also unaffected by the loss of FAK (Figure S1C). Similarly, ROS production in response to *Salmonella* was detected at equivalent levels in both WT and FAK-deficient macrophages (Figure S1D). As expected, activation of NF-κB was completely dependent upon expression of the Toll-like receptor TLR4 (Figure S1E)

In contrast to the rapid activation of ERK, p38 and NF-κB, activation of FAK (as measured by autophosphorylation at Y397) was detected later, at 5 h post-infection (Figure 1B) suggesting that FAK activation is triggered by intracellular bacteria. FAK activation correlated temporally with its recruitment to the surface of SCVs (marked by staining for the lysosomal membrane protein LAMP-1, Figure 1C and D). At 5 h post-infection approximately 75% of LAMP-positive SCVs were also positive for FAK (Figure 1C and D). In contrast, FAK was rarely detected on phagosomes containing either LPS-coated beads (Figure 1 E and F) or the nonpathogenic *Escherichia coli* strain DH5α (Figure 1G and H) at any time point examined, although they were strongly positive for LAMP-1. These observations suggest that FAK activation and recruitment to SCVs occurs in response to the Salmonella SPI-2 system, and is not simply a result of bacterial recognition by TLR4 or other pattern recognition receptors.

### The *Salmonella* SPI-2 apparatus is required for FAK activation

**Figure 2:**
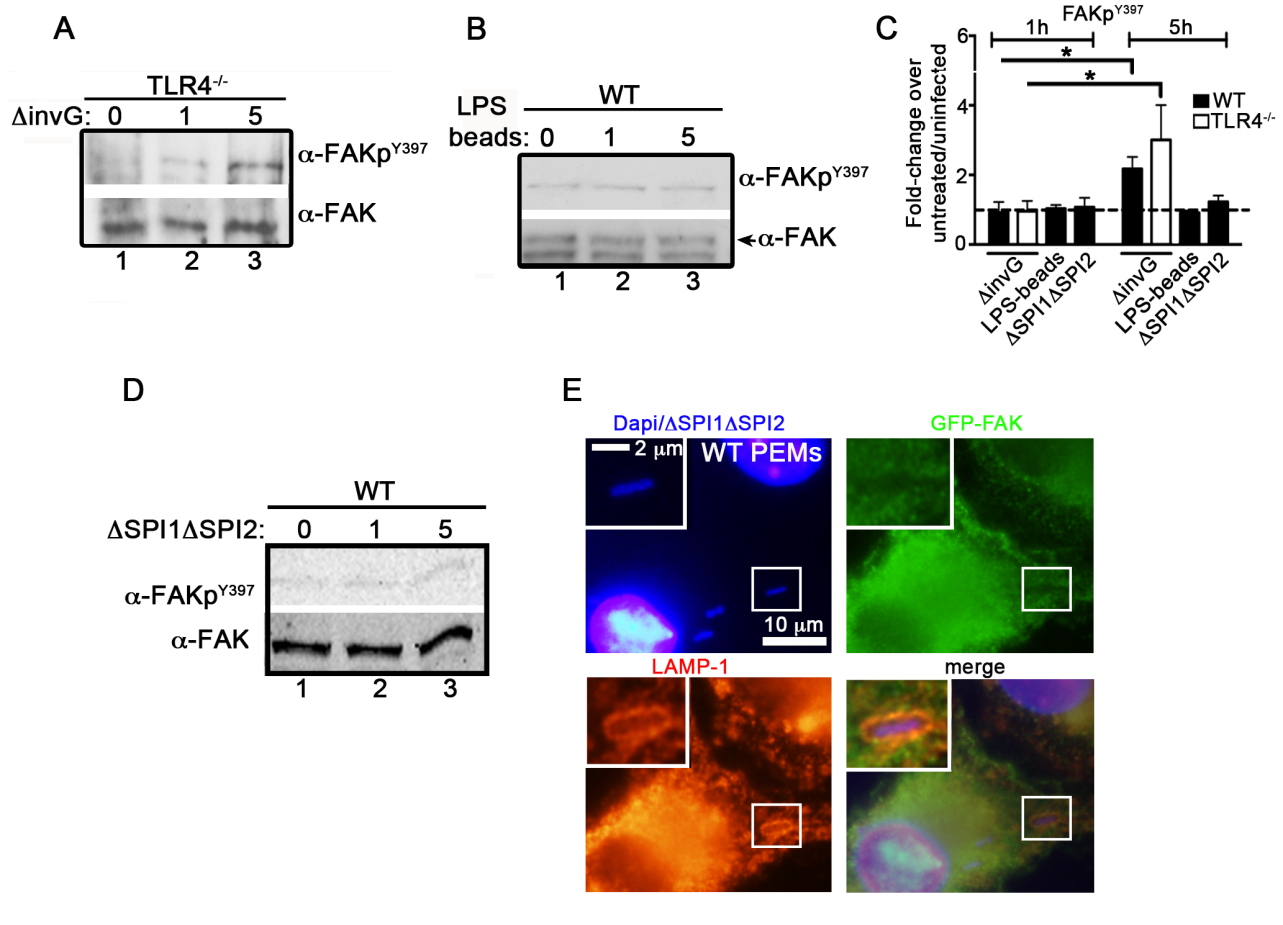
*Salmonella* specifically targets FAK for activation. (A) TLR4^−/−^ PEMs were incubated with *ΔinvG Salmonella* for 0–5 hours before immunoblotting with the indicated antibodies. (B) WT PEMs were incubated with LPS-coated beads for 0–5 hours before immunoblotting for the indicated antibodies. (C) Levels of phosphorylated proteins were quantified by densitometry, normalized to the amount of total protein present in each sample, and expressed relative to the basal level in uninfected cells. Values are means ± SEM, N = 3, *p<0.05. Dashed line drawn at 1 indicates basal levels of activation. (D) WT PEMs were incubated with the *orgA::tet*; *spiA::kan Salmonella* strain deficient in both the SPI1 and SPI2 T3SSs (ΔSPI1ΔSPI2) for 0–5 hours before immunoblotting with the indicated antibodies. (E) WT PEMs expressing GFP-FAK were incubated with ΔSPI1ΔSPI2 *Salmonella* for a total of 5 hours before analysis by immunofluorescence. Cells were co-stained with antibodies recognizing LAMP1 (red). DAPI was used to visualize nuclei and bacteria (blue). Bars represent 10 µm or 2 µm in enlarged inset.

To test this hypothesis, we examined the ability of *Salmonella* to activate FAK in macrophages isolated from TLR4^−/−^ mice (Figure S2A). As shown in Figure 2A, incubation of TLR4^−/−^ PEMs with *ΔinvG Salmonella* did not impair FAK activation at the 5 h time point. Conversely, FAK was not activated in WT macrophages at any time point by LPS-coated beads (Figure 2B and C), although p38 was robustly activated under these conditions (Figure S2B). Together, these observations indicate that FAK activation by *Salmonella* is not mediated by activation of TLR4.

We next examined whether the activation of FAK was dependent on the *Salmonella* SPI-2 T3SS. To do this, we utilized a Salmonella strain deficient in both the SPI1 and SPI2 T3SSs (ΔSPI1ΔSPI2; *orgA::tet*; *spiA::kan*) [32]. Importantly, incubation of WT PEMs with ΔSPI1ΔSPI2 *Salmonella* did not result in significant activation of FAK, nor was FAK recruited to ΔSPI1ΔSPI2-containing SCVs (Figure 2C, D and E). Similar results were observed in WT PEMs incubated with the *E. coli* strain DH5α, which stimulates TLR4 but lacks effector driven manipulation of the host (Figure S2C–D). Together, these data indicate that the *Salmonella* actively manipulates FAK in a SPI-2-dependent manner, as LPS-coated beads, *E. coli* and *Salmonella* deficient in both the SPI-1 and SPI-2 T3SS were incapable of driving FAK activation.

### Activation of FAK promotes Akt activity

**Figure 3:**
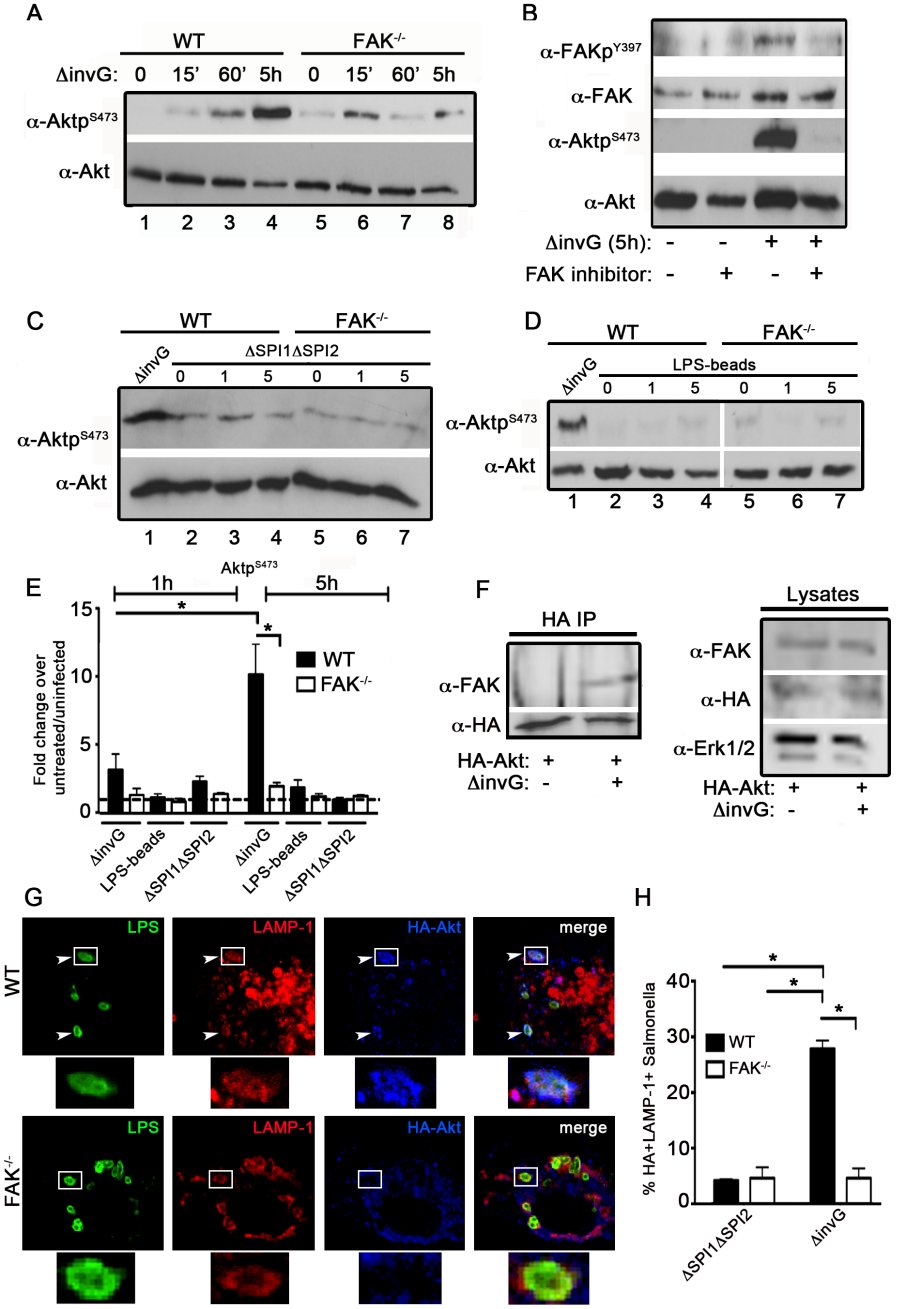
Akt activation in response to *Salmonella* requires FAK. (A) WT and FAK^−/−^ PEMs were incubated for 0–5 hours with *ΔinvG Salmonella* before immunoblotting with the indicated antibodies. (B) WT PEMs were pretreated for 1 hour with the FAK-kinase inhibitor PF-228 (0.5 µm) before incubation with *ΔinvG Salmonella* for 5 hours. Lysates were immunoblotted with the indicated antibodies. (C–D) WT or FAK^−/−^ macrophages were incubated with either the ΔΣΠΙ1ΔΣΠΙ2 strain (C) or LPS-coated beads (D) for 0–5 hours before immunoblotting with the indicated antibodies. In panels C and D, WT PEMs were also incubated with *ΔinvG* (5 h) for comparison of Akt activation. Vertical white lines in panel D indicate non-contiguous lanes generated from a single exposure. (E) Levels of phosphorylated proteins were quantified by densitometry, normalized to the amount of total protein present in each sample, and expressed relative to the basal level in uninfected cells. Values are means ± SEM, N = 3, *p<0.05. Dashed line drawn at 1 indicates basal levels of activation. (F) WT PEMs expressing HA-Akt were either left untreated or incubated with *ΔinvG Salmonella* for 5 hours. Lysates were subjected to immunoprecipitation (IP) with anti-HA antibodies before immunoblotting for FAK and HA. In parallel, lysates were immunoblotted for FAK, HA and ERK1/2 to control for the level of expression. (G) WT and FAK^−/−^ macrophages expressing HA-Akt were stained with anti-HA and anti-LAMP1 before analysis by confocal microscopy. White boxes show enlarged regions in lower panel. Arrows indicate HA+LAMP+ *Salmonella* evident in WT PEMs. (H) The percentage of ΔΣΠΙ1ΔΣΠΙ2 (first grouping) or *ΔinvG* (second grouping) *Salmonella* co-localizing with HA and LAMP-1 5 h post-infection. At least 100 bacteria were counted per condition. Values are means ± SEM, N = 3, *p<0.05.

Because FAK activation can stimulate the downstream activation of Akt [28], we next examined Akt activity using phospho-specific antibodies. As shown in Figure 3A, the increase in FAK activity observed in Figure 1B was paralleled by a robust increase in the activation of Akt. In contrast to the ERK, p38 and NFκB pathways, which were unaffected by FAK knockout (Figure S1A–B), Akt activation was severely attenuated in the absence of FAK. Furthermore, bacterially induced Akt activation was dependent upon FAK kinase activity, as it was nearly abrogated by treatment of cells with a FAK-specific kinase inhibitor (PF-228) [33] (Figure 3B). Similar to FAK, Akt activation required SPI-2, as it was not observed in cells incubated with the ΔSPI1ΔSPI2 mutant (Figure 3C and E), LPS-coated beads (Figure 3D–E) or *E. coli* (Figure S2E–F).

As Akt is known to physically interact with FAK in response to external stimuli [34], we next determined if this interaction is stimulated in the context of infection. Co-precipitation of FAK with Akt was not detectable in uninfected cells, but was readily observed upon incubation with *ΔinvG Salmonella* (Figure 3F). We also found that Akt associates with the membranes of SCVs in WT macrophages at 5 h post-infection, but not in FAK-deficient cells (Figure 3G–H). Furthermore, Akt localization to the SCV is driven by components of the SPI-2 system as we observed little recruitment of Akt to LAMP-1-positive vesicles in cells treated with the ΔSPI1ΔSPI2 strain (Figure 3H). Taken together, these data suggest that Akt is recruited to SCVs in a FAK-dependent manner, and that coordinate activation of both FAK and Akt requires elements of the SPI-2 T3SS machinery.

### Pathogen-induced LC3 recruitment is enhanced in FAK^−/−^ macrophages

**Figure 4:**
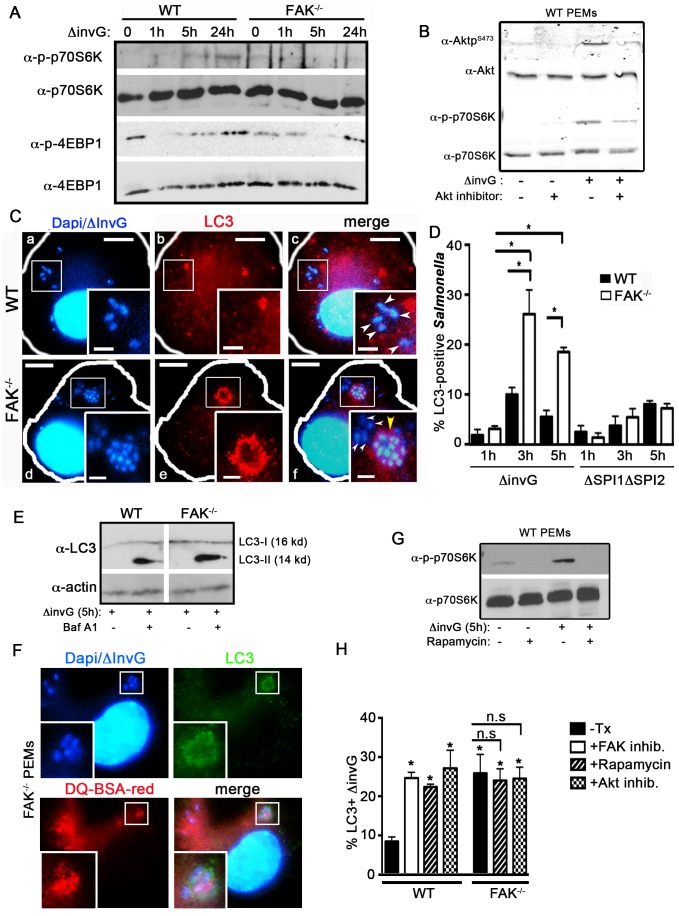
FAK-dependent Akt-mTOR signaling inhibits the recruitment of LC3 to SCVs. (A) WT and FAK^−/−^ PEMs were incubated with *S. typhimurium* strain *ΔinvG* for 0–24 hours before immunoblotting with the indicated antibodies. (B) WT PEMs were incubated with Akt inhibitor AKTV/triciribine (10 µm) for 30 minutes before infection with *S. typhimurium* strain *ΔinvG* for 5 hours. Lysates were immunoblotted with the indicated antibodies. (C) WT and FAK^−/−^ PEMs were incubated with *S. typhimurium* strain *ΔinvG* for a total of 5 hours before analysis with antibodies recognizing LC3. DAPI was used to visualize nuclei and bacteria. Bars represent 10 µm. White boxes show enlarged regions in inset panels, bars represent 2 µm. White arrowheads indicate LC3-negative bacteria, yellow arrow heads indicate LC3-positive bacteria (panels c, f). Yellow arrowhead in panel f denotes an LC3+ cluster of bacteria. (D) The percentage of *ΔinvG* (first grouping) or ΔΣΠΙ1ΔΣΠΙ2 (second grouping) *Salmonella* co-localizing with LC3 at the indicated time points is displayed. At least 100 bacteria were counted per condition per time point. Values are means ± SEM, N = 3, *p<0.05. (E) WT and FAK^−/−^ PEMs were incubated with *S. typhimurium* strain *ΔinvG* for 5 h with or without Bafilomycin A1 (300 ng) before immunoblotting with the indicated antibodies. Vertical white line indicate non-contiguous lanes generated from a single exposure. (F) FAK^−/−^ PEMs were incubated with DQ-BSA-red for 1 hour before infection with *S. typhimurium* strain *ΔinvG* for 5 h. Cells were co-stained for LC3 and DAPI was used to visualize nuclei and bacteria. White boxes show enlarged regions in inset panel. (G) WT PEMs were pretreated with the mTORC1 inhibitor rapamycin (4 µm) for 1 hour before incubation with *ΔinvG Salmonella* for a further 5 hours. Cells were lysed before immunoblotting with the indicated antibodies. (H) WT and FAK^−/−^ PEMs were pretreated with rapamycin (4 µm), Akt inhibitor AKTV/triciribine (10 µm) or left untreated (-Tx) or before infection with *S. typhimurium* strain Δ*invG* for a further 5 hours. WT macrophages were also pretreated with the FAK inhibitor PF228 (0.5 µm) for 1 hour prior to incubation with *ΔinvG Salmonella* for 5 hours. Cells were then assessed for the percentage of *ΔinvG Salmonella* co-localizing with LC3. At least 100 bacteria were counted per condition. Values are means ± SEM, N = 3, *p<0.05. N.s. not significant.

Because the PI3K/Akt pathway functions as a critical upstream regulator of mTOR signaling, we next determined whether the mTOR pathway was affected by the loss of FAK by examining the activation of two major mTOR targets, p70S6K and 4EBP1. In WT cells, incubation with *ΔinvG* Salmonella induced the activation of p70S6K 5 h post-infection and this was sustained throughout a 24 h time-course, whereas this response was completely abrogated in FAK^−/−^ cells (Figure 4A). In addition, pharmacological inhibition of Akt with the specific inhibitor AKTV/triciribine in WT cells attenuated bacterially induced phosphorylation of p70S6K, confirming that the mTOR pathway is activated downstream of Akt in response to *Salmonella* (Figure 4B). Interestingly, infection with *ΔinvG Salmonella* resulted in the strong downregulation of 4EBP1 phosphorylation, as previously reported in epithelial cells [34]. This downregulation was evident in both cell types, although the kinetics of deactivation appeared to be delayed in FAK-deficient cells (Figure 4A). Nonetheless, phospho-4EBP1 returned to basal levels in WT and FAK^−/−^ cells after 24 h (Figure 4A). Taken together, these data suggest that in response to the presence of intracellular *Salmonella*, FAK may be required for Akt-mediated activation of mTOR, and that this leads to the activation of p70S6K but not 4EBP.

Given that mTOR signaling is attenuated in the absence of FAK, we next investigated whether this resulted in the capture of *Salmonella* by autophagy or an autophagy-like process. In epithelial cells, the SPI-1 TTSS damages SCVs, resulting in the exposure of the SCV lumen to the cytosolic galectins-3, 8 and 9, rapid ubiquitylation and recruitment of the autophagic adaptor proteins p62, NDP52 and optineurin [21], [22], [35]. This is followed by the conjugation of LC3 and ultimately the formation of an autophagosome-like double membrane [17], [18], [36]. In macrophages, we found that virtually all internalized bacteria became LAMP-1 positive within 1 h of infection, indicating that SCVs formed efficiently in both WT and FAK-deficient cells (Figure S3). However, whereas the ubiquitin adaptor p62 was readily detected around SCVs in HeLa cells infected with the invasive WT *Salmonella* strain SL1344 (Figure S4A), in macrophages, SCVs containing non-invasive (*ΔinvG*) *Salmonella* were not associated with either p62 or ubiquitin (Figure S4B–C). In addition, it has previously been shown that mTOR is recruited to SCVs in epithelial cells several hours after infection with WT Salmonella [37], which we confirmed in Hela cells (Figure S5). However, despite numerous attempts with multiple mTOR antibodies and fixation conditions, we were unable to detect mTOR on either lysosomes or SCVs in mouse macrophages.

We next examined the recruitment of LC3, which is conjugated onto the membrane of autophagosomes and is a well-characterized marker of these structures [5]. Despite the absence of detectable p62 and ubiquitin, LC3 was found to associate with *ΔinvG Salmonella* in both WT and FAK^−/−^ PEMs (Figure 4C). However, the fraction of intracellular bacteria co-localized with LC3 was significantly higher in FAK-deficient cells: at 3 hours post-infection 25% of internalized bacteria were in LC3-positive structures, vs. 10% for WT cells (Figure 4C–D) and this ratio was maintained throughout the 5-hour time course (Figure 4D, first grouping). Furthermore, LC3 recruitment in FAK^−/−^ macrophages was dependent on the SPI-2 system since we did not observe LC3 localization with either the ΔSPI1ΔSP2 strain or *E. coli* (Figure 4D, second grouping and Figure S6A–B). Enhanced levels of the mature, lipid-conjugated form of LC3 (LC3-II) were also observed in FAK-deficient macrophages 5 hours post-infection compared to WT cells (Figure 4E). In addition, LC3 often co-localized with LAMP1 around clusters of multiple bacteria, a phenotype that was not observed in WT cells (Figure 4C and S6C). This is in stark contrast to the autophagy of SPI-1-competent *Salmonella* in epithelia, which has previously been observed to occur early and transiently, with little co-localization between bacteria and LC3 evident beyond 2 hours post-infection [18].

The fusion of autophagosomes with lysosomes is the final stage of autophagy. To monitor lysosomal activity within LC3-positive compartments, cells were assayed for their ability to process DQ-BSA (a derivative of BSA that fluoresces red only when cleaved by proteolytic enzymes). As shown in Figure 4F, red fluorescent BSA colocalizes with LC3-positive *ΔinvG* Salmonella 5 hours post-infection, indicating that, unlike conventional SCVs, these compartments are functionally capable of bacterial degradation.

Finally, we investigated whether the LC3 translocation observed in the absence of FAK was a direct consequence of reduced mTORC1 signaling. To examine this, macrophages infected with *ΔinvG Salmonella* were pre-treated with the FAK kinase inhibitor PF-228, an Akt specific inhibitor (AKTV/triciribine) or rapamycin to down-regulate mTORC1 activity (Figure 4B and 4G) prior to staining for LC3. In WT cells, inhibition of mTOR signaling resulted in a significant increase in the percent of *Salmonella* associated with LC3, rising from 8% in untreated cells to 25–30% in inhibitor-treated cells (Figure 4H and S7A). As expected, rapamycin and AKTV had no effect on LC3 recruitment in FAK^−/−^ macrophages since FAK deficiency already results in down-regulated mTORC1 signaling (Figure 4H and S7B). Thus, pharmacological inhibition of the FAK/Akt/mTORC1 signaling pathway phenocopies the enhanced recruitment of LC3 to SCVs observed in FAK-deficient macrophages, suggesting that FAK signaling suppresses the autophagic response to intracellular *Salmonella*.

### Atg5 and ULK1 are required for pathogen-induced LC3 recruitment

**Figure 5:**
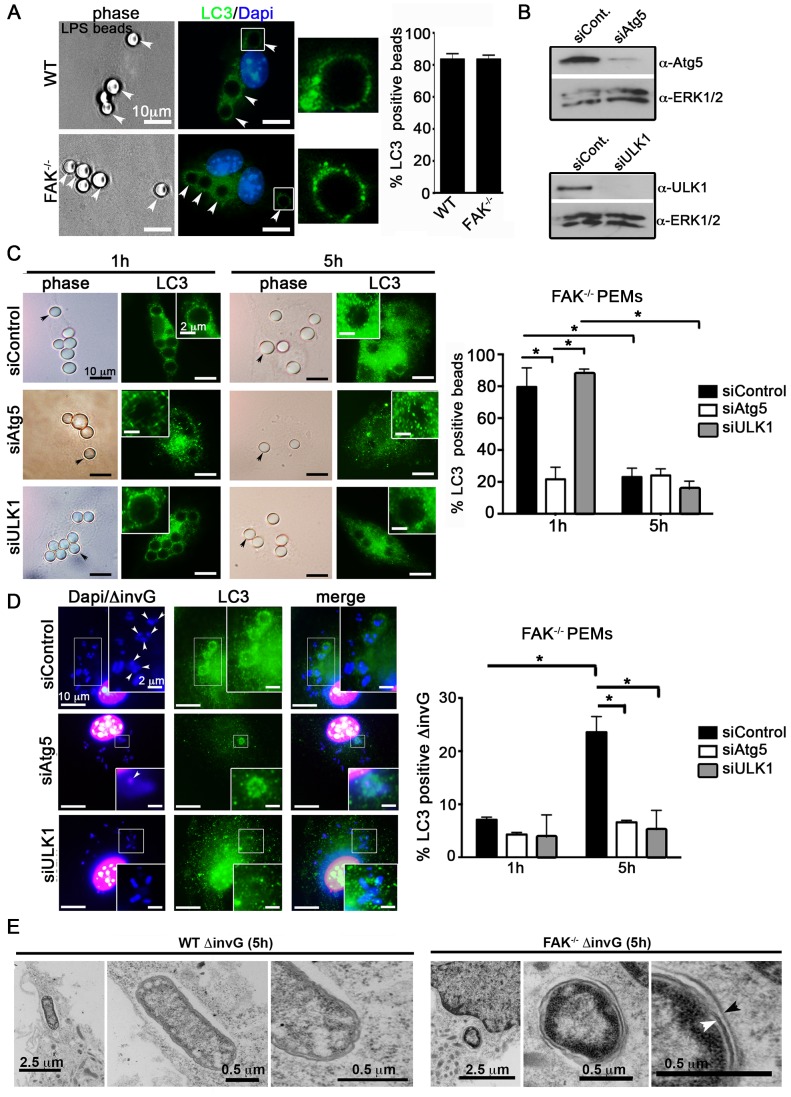
SPI-1-deficient *Salmonella* are targeted by autophagy in the absence of FAK. (A) WT and FAK^−/−^ PEMs were incubated with LPS-coated beads (50∶1; beads∶cell) for 1 hour before analysis with antibodies recognizing LC3. DAPI was used to visualize nuclei. Bars represent 10 µm. The percentage of LPS-beads co-localizing with LC3 is displayed to the right. At least 100 beads were counted per condition. Values are means ± SEM, N = 3. (B) PEMs were treated with Atg5, ULK1 or control siRNAs for 48 hours before immunoblotting with the indicated antibodies. (C–D) FAK^−/−^ PEMs treated with Atg5, ULK1 or control siRNA for 48 hours were incubated with LPS-coated beads or *ΔinvG Salmonella* for 1 or 5 hours before analysis with antibodies recognizing LC3. The percentage of LPS-beads or *Salmonella* co-localizing with LC3 is displayed to the right. At least 100 beads or bacteria were counted per condition. Values are means ± SEM, N = 3. (E) WT and FAK^−/−^ PEMs were infected with *ΔinvG* for 5 hours before preparation and examination by transmission electron microscopy (TEM). Arrowheads indicate double membrane.

Recent studies have shown that components of the autophagy system are capable of capturing pathogens inside vacuolar compartments, bypassing several steps central to canonical autophagy [5]. For example, LC3-assisted phagocytosis (LAP) is an Atg5-dependent process whereby LC3 is rapidly (<1 h) recruited to phagosomes independent of ULK-mediated isolation membrane biogenesis [10], [36], [38]. To determine if LC3 recruitment to SCVs occurs by LAP we compared *ΔinvG* Salmonella to LPS-coated beads, a well-established driver of LAP [7], using several criteria. First, 80% of LPS-coated beads became LC3-positive within 1 h in WT macrophages, and this was not dependent on FAK (Figure 5A). In comparison, only 8% of bacteria were LC3-positive at this time point (Figure 5D). Second, recruitment of LC3 to bead-containing phagosomes decreased over time, while recruitment to intracellular Salmonella increased (compare Figure 5C to 5D). Third, LC3 recruitment to bead-containing phagosomes was dependent upon Atg5 but not ULK1 (Figure 5C), while recruitment to SCVs required both Atg5 and ULK1 (Figure 5D). Fourth, LAP has been reported to require production of ROS [39], and we found that LC3 recruitment to SCVs occurred normally in macrophages derived from mice genetically deficient in the NADPH oxidase subunit gp91-phox (gp91^phox−/−^), after treatment with the FAK inhibitor PF-228 (Figure S8). Finally, transmission electron microscopy (TEM) revealed that a significant fraction of intracellular Salmonella in FAK-deficient macrophages were contained within the double membrane characteristic of true autophagosomes, while such structures were only rarely observed in WT macrophages (Figure 5E). Together, these data strongly suggest that disruption of the FAK/Akt/mTORC1 signaling pathway leads to autophagic capture of bacteria, rather than LAP.

### FAK deficiency protects mice from lethal *S. typhimurium* infection

**Figure 6:**
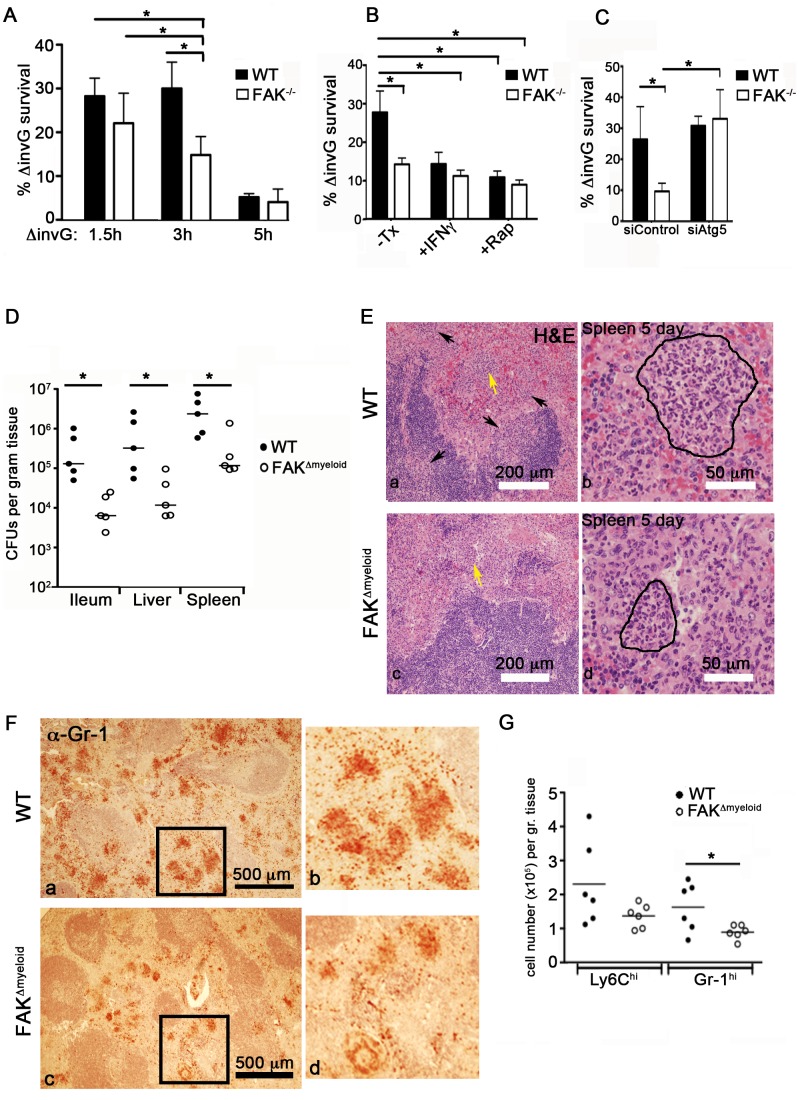
FAK deficiency improves control of *Salmonella* infection. (A) WT and FAK^−/−^ PEMs were incubated with *S. typhimurium* strain *ΔinvG* for 1.5, 3 and 5 hours. Bacterial survival was then assayed using a standard gentamycin resistance assay described in the Materials and Methods. Values are means ± SEM, N = 6, *p<0.05. (B) WT and FAK^−/−^ PEMs were pretreated with IFN-γ (50 ng/ml), rapamycin (4 µm), or left untreated before incubation with *ΔinvG Salmonella* for 3 hours. Bacterial survival was then assayed as described in (A). Values are means ± SEM, N = 6, *p<0.05. (C) PEMs were treated with control siRNA or depleted of Atg5 before incubation with *ΔinvG Salmonella* for 3 hours. Bacterial survival was then assayed as described in (A). Values are means ± SEM, N = 3, *p<0.05 vs WT siControl. (D) Bacterial loads in WT (black circles) and FAK^Δmyeloid^ mice (white circles) in the indicated tissues 5 days after oral infection with *S. typhimurium* strain SL1344. Each point indicates data from an individual mouse. *p<0.05. (E) Hematoxylin and eosin (H&E) staining of spleen isolated from WT and FAK^Δmyeloid^ mice 5 days post-infection with *S. typhimurium* strain SL1344. Arrows indicate areas of leukocyte infiltration. Lesion indicated by yellow arrow is outlined in the higher magnification panel to the right. Bars represent 200 µm and 50 µm. (F) Gr-1 immunostaining of spleens isolated from WT and FAK^Δmyeloid^ mice 5 days post-infection with *S. typhimurium* strain SL1344. Panels b and d are enlarged sections indicated by black boxes in panels a and c. Gr-1-positive cells appear red-brown. (G) Quantitation of flow cytometric data collected from the spleens of infected mice. *p<0.05.

To determine whether the down-regulation of Akt-mTOR signaling observed in FAK^−/−^ cells affects *Salmonella* survival, WT and FAK^−/−^ PEMs were incubated with *S. typhimurium* for 30 minutes, treated with gentamycin to kill extracellular bacteria, and intracellular bacterial survival was quantified after a further 1, 2.5 and 4.5 hours of infection. While the loss of FAK did not affect the initial uptake of bacteria by macrophages (Figure S9), survival of intracellular bacteria was reduced by 50% in FAK^−/−^ PEMs at the 3 hour time point, relative to WT controls (Figure 6A). By 5 hours post-infection, bacterial survival was reduced to statistically similar levels in WT and FAK^−/−^ macrophages, which may be the result of up-regulated MyD88-dependent inflammatory signaling occurring in both cell types (Figure S1). In addition, treatment with the TH1 cytokine IFN-γ or rapamycin to stimulate autophagy greatly increased the killing ability of WT cells, reducing bacterial survival from 30% in untreated cells to 15% in IFN-γ treated macrophages and 12% in rapamycin-treated cells (Figure 6B).

To determine whether the inhibition of autophagy in FAK^−/−^ cells could overcome the enhanced killing capacity of these cells, PEMs were depleted of Atg5 and the bactericidal activity of these cells was assessed. As shown in Figure 6C, Atg5 depletion restored bacterial survival to WT levels in FAK-deficient cells. These data, together with the kinetics of LC3 association with SCVs suggest that the activation of FAK by the Salmonella SPI-2 system suppresses an autophagic process distinct from LAP that is critically important for limiting intracellular bacterial growth.

Given the difference in bactericidal activity (Figure 6A–C) and enhanced autophagic targeting of *Salmonella* observed in FAK^−/−^ PEMs (Figure 5), we next examined the role of macrophage-specific FAK in the overall host response to *Salmonella i*nfection. WT or FAK^Δmyeloid^ mice were orally inoculated with virulent *S. typhimurium* (SL1344) and the colonization of tissues was examined. As shown in Figure 6D, after 5 days of infection, bacterial levels were 10–50-fold higher in the ileum, liver and spleen of WT mice compared to FAK-deficient mice. In addition, reduced bacterial burdens in FAK^Δmyeloid^ animals correlated with a significant attenuation of tissue pathology. Histological examination of spleens isolated from FAK^Δmyeloid^ mice 5 days post-infection showed reductions in the size and number of granulomatous lesions (Figure 6E). Furthermore, immunohistochemical staining revealed lower numbers of Gr-1-positive neutrophils in the spleens of FAK-deficient animals (Figure 6F). Consistent with these observations, flow cytometric analysis showed reduced numbers of both Ly6C^hi^ monocytes and Gr-1-positive neutrophils in the spleens of infected animals (Figure 6G). Together, these data indicate that the loss of FAK from macrophages enhances the clearance of S. *typhimurium in vivo*, and attenuates the inflammatory response to infection.

## Discussion

Autophagy is a dynamic process that broadly impacts multiple immunological functions. At the level of innate immunity, autophagy is a strategy employed by host cells for the capture and elimination of intracellular pathogens (xenophagy). Not surprisingly, successful pathogens have evolved numerous ways to protect themselves from autophagic degradation. Recent evidence indicates that *Salmonella* can actively suppress autophagy to promote its intracellular survival in epithelial cells [37]. Here we show that *S. typhimurium* also inhibits autophagic processing in macrophages, through a pathway mediated by focal adhesion kinase and its downstream signaling partner Akt.

### Role of the SPI-2 machinery in autophagy suppression

The SPI-1-encoded invasion machinery is used by *Salmonella* to actively infect intestinal epithelial cells, which are not inherently phagocytic. It is now well established that SPI-1 components also trigger an early autophagic response by damaging the membrane of the SCV [18], [23], resulting in ubiquitination of SCV components and the recruitment of adaptor proteins such as p62, NDP52 and optineurin that couple the SCV to the autophagy machinery [21], [22], [40]. In addition, a fraction of bacteria escape the vacuole and become ubiquitylated in the cytosol [18].

However, after penetrating the intestinal epithelium, SPI-1 is down-regulated and bacteria that have reached the submucosa can be captured by macrophages or other phagocytic cells [2], [41]. Entry of bacteria into the phagosomal environment triggers expression of the SPI-2 machinery, which reaches maximal levels 2–5 h post-internalization, and is critical to intracellular survival [1]. To distinguish between SPI-1- and SPI-2-mediated events, we made use of the mutant strain *ΔinvG*, which lacks a functional SPI-1 secretion system.

Here we show that FAK is recruited to the SCV and activated in a manner that is dependent upon the SPI-2 T3SS. Neither recruitment nor activation was observed after incubation of macrophages with another Gram-negative microbe, *E. coli*, or LPS-coated beads, indicating that activation of TLR4 is insufficient to drive FAK activation. A more direct demonstration is provided by the observation that a strain of *Salmonella* deficient in both the SPI-1 and SPI-2 T3SS failed to recruit or activate FAK. It is also noteworthy that FAK activation peaks in parallel with expression of the SPI-2 T3SS, at 2–5 hours post infection [42]. At present however, it is still unclear which SPI-2 effector(s) are responsible for manipulating FAK activity. Of the nearly 30 secreted SPI-2 molecules, less than half have an assigned function [1]. While unraveling which effector or combination of effectors promotes FAK activation is likely to be complex, this will be an important area of future investigation.

### Akt links FAK to mTORC1

**Figure 7:**
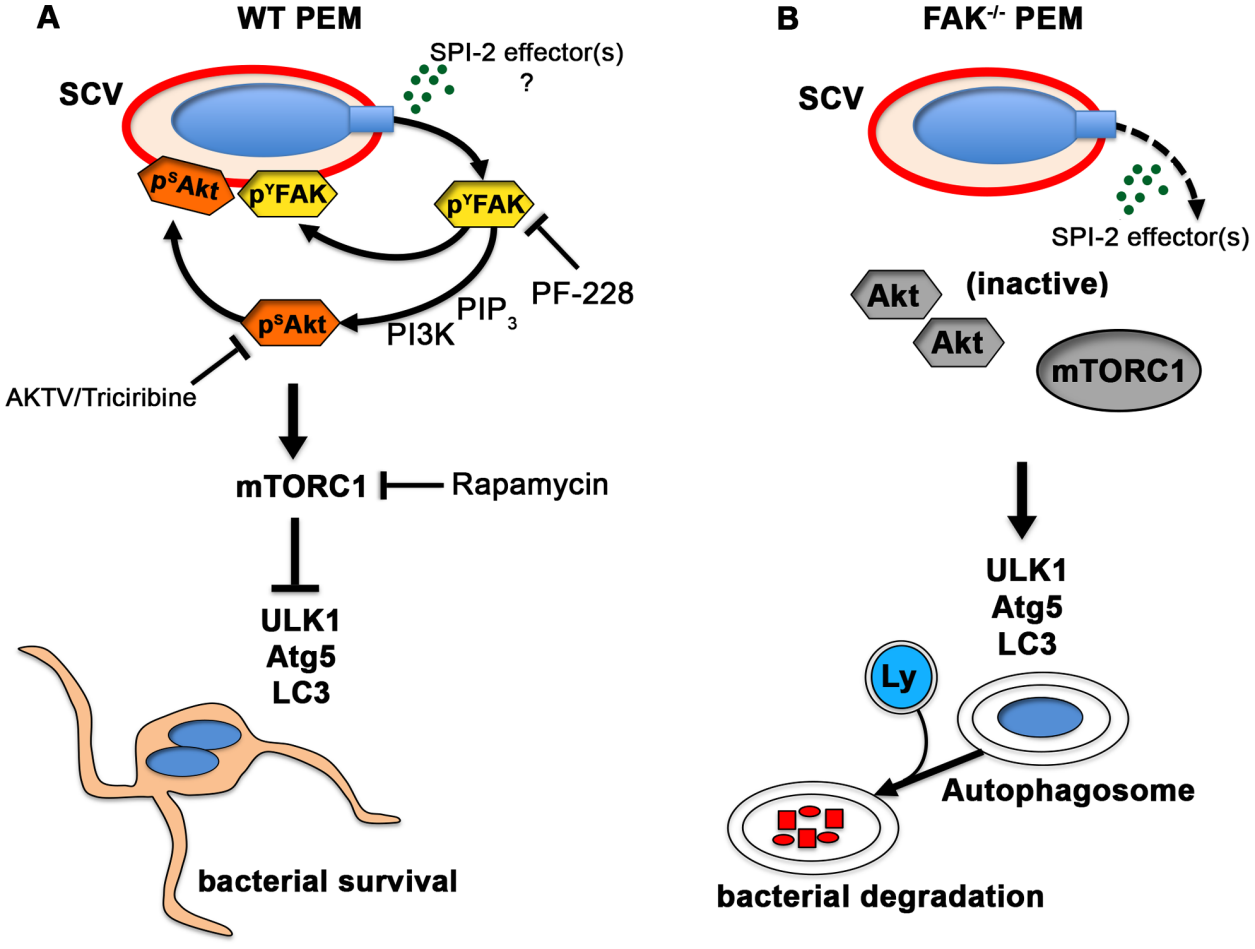
The *Salmonella* SPI-2 system triggers FAK activation and suppresses the autophagic capture of intracellular *Salmonella*. (A) In WT PEMs, SPI-2-compentent *Salmonella* target FAK-Akt signaling for activation and recruitment to the SCV membrane. The increase in FAK and Akt activity is paralleled by a robust increase in the activation of mTORC1, which suppresses the autophagic response and promotes bacterial survival. Phamacological inhibition of FAK (PF-228) or Akt (AKTV/triciribine) inhibited LC3 recruitment to *ΔinvG* SCVs, whereas inhibition of mTORC1 (rapamycin) enhanced LC3 colocalization with bacteria, phenocopying conditions observed in FAK-deficient macrophages. (B) In the absence of FAK, Akt remains inactive and is unable to activate mTORC1. LC3 is more efficiently recruited to SCVs resulting in autophagosome formation and the enhanced elimination of intracellular bacteria. Ly, lysosome.

Akt serves as a major signaling hub for numerous cell processes, including cell survival and proliferation. Akt is activated during invasion of epithelial cells in response to the SPI-1 T3SS effector SopB/SigD, and while it is not required for bacterial entry, it appears to suppress the apoptosis of infected cells [43], [44], [45]. Akt has also been linked to intracellular *Salmonella* growth [46], but its mechanism of action in this context is not well understood. Here we show that, in macrophages, Akt activation parallels that of FAK, and is similarly SPI-2-dependent, suggesting that it occurs independently of SopB. We also show that knockout or pharmacological inhibition of FAK impairs bacterially-induced Akt activation, resulting in reduced mTORC1 signaling and enhanced autophagic clearance of intracellular bacteria (Figure 7). Together these findings suggest that *Salmonella* utilize one or more SPI-2 effectors to evade autophagic capture in macrophages by targeting the FAK-Akt-mTORC1 signaling cascade (Figure 7A), resulting in enhanced intracellular survival. *In vivo*, selective knockout of FAK in macrophages also resulted in significantly reduced bacterial burdens and inflammation after oral infection. However, whether these differences in susceptibility were directly due to an enhanced cell-autonomous autophagic response is currently unknown, and is the topic of ongoing research.

### Loss of FAK accelerates autophagosome formation

Recent studies suggest that autophagy can also take place in the absence of canonical autophagy components. For example, Collins et al [47] reported that although *Mycobacterium marinum* is ubiquitinated and targeted by autophagy in a double-membrane bound phagosome, this process does not require the function of Atg5. Conversely, in LC3-assisted phagocytosis (LAP), engagement of TLRs with their cognate ligands leads to the non-canonical recruitment of Atg proteins to a single-membrane bound phagosome in a process that requires Atg5 but not ULK1-mediated phagophore formation [48]. We observed LC3 associated with ∼10% of bacteria in wild type cells, increasing to 30% in FAK-deficient macrophages. Furthermore, we found that association of LC3 with intracellular *Salmonella* required both Atg5 and ULK1, which, in FAK-deficient cells, resulted in the containment of bacteria within multilamellar structures resembling canonical autophagosomes. Previous studies have shown that pharmacological induction of autophagy with the mTORC1 inhibitor rapamycin does not affect TLR-promoted LAP [10]. Importantly, we found that rapamycin treatment significantly increased the percent of LC3-positive *Salmonella* in WT macrophages whereas rapamycin treatment had no effect on FAK^−/−^ macrophages since FAK deficiency already results in inhibition of mTORC1 signaling. However, autophagosome formation in FAK^−/−^ macrophages did not require ubiquitylation or the recruitment of p62. Nonetheless, in the absence of FAK, bacterial survival was significantly reduced, and could be restored to WT levels by depletion of Atg5. These results highlight the fact that components of the autophagy machinery play important roles in immune regulation that are distinct from both canonical autophagy and LAP.

It has also recently been demonstrated that signals other than ubiquitin can initiate antibacterial autophagy. Shahnazari and colleagues [49] showed that the lipid second messenger diacylglycerol (DAG) localizes with LC3+ *Salmonella* and that inhibition of DAG formation impairs the autophagy of bacteria. While local production of DAG did require SPI-1 activity, this observation raises the possibility that an alternate adaptor may facilitate the recruitment of LC3 to SPI-1 deficient *Salmonella*.

In summary, we demonstrate that intracellular *Salmonella* actively suppress autophagy through a novel SPI-2-dependent mechanism that involves the recruitment of FAK to the SCV. FAK activation triggers a downstream signaling cascade involving Akt and mTORC1 that enhances intracellular survival by attenuating the autophagic response.

## Materials and Methods

### Ethics statement

All experiments in this study were performed in strict accordance with the recommendations in the Guide for the Care and Use of Laboratory Animals of the National Institutes of Health. Protocols were approved by the Institutional Animal Care and Use Committee at the University of Virginia (Protocol number 3488). All efforts were made to minimize animal suffering during the course of these studies.

### Mice

The generation of myeloid-specific conditional FAK knockout mice and their control littermates have been described previously [26]. TLR4 knockout mice were purchased from Jackson Laboratories (stock # 007227) and gp91^phox−/−^ mice were a kind gift from Dr. Borna Mehrad, University of Virginia, Charlottesville, VA. Mice were kept in pathogen-free conditions and allowed free access to food and water.

### Cell culture

Peritoneal macrophages (PEMs) were isolated from mouse peritoneal lavage fluid using sterile PBS supplemented with 0.5% BSA and 2 mM EDTA. Macrophages were seeded onto petri dishes and cultured in alpha minimal essential medium (αMEM; Gibco) containing 10% heat inactivated fetal bovine serum (FBS), 1% pen/strep and 10% L929-conditioned media as a source of colony stimulating factor-1 (CSF-1). Hela cells were cultured in Dulbecco's modified Eagle medium (DMEM; Gibco) supplemented with 10% heat inactivated FBS, 1% L-glutamine and 1% pen/strep.

### Bacterial strains and culture

*Salmonella typhimurium* strain SL1344, the isogenic invasion deficient mutant Δ*invG* (SPI-1-deficient) and the *orgA::tet*, *spiA::kan* (double SPI1 and SPI2 mutant/ΔSPI1ΔSPI2) strain have been described previously [32], [50], [51]. Bacteria were grown under invasion-inducing conditions as described by Lee and Falkow (1990) [52]. Briefly, a single colony was inoculated into LB broth and grown for 8 h under aerobic conditions and then diluted back to 1∶1000 under oxygen-limiting conditions (i.e., not shaken) for overnight growth. Bacteria were harvested the following morning when their density reached ∼5×10^8^ to 7×10^8^ CFU/ml. In experiments using non-pathogenic *Escherichia coli* strain DH5α, a single colony was inoculated into LB and grown under aerobic conditions overnight before use.

### Gentamycin resistance assay

The gentamicin resistance assay has been described previously [53]. Briefly, cells were seeded at 1–2×10^5^ cells/well onto 24-well culture dishes 18 h prior to infection. For some experiments, PEMs were treated with 50 ng/ml IFN-γ (PeproTech) overnight or incubated for 1 hour prior to infection with 4 µM rapamycin (Calbiochem). Cells were then infected in complete media without antibiotics at a multiplicity of infection (MOI) of 100 for 30 min with *ΔinvG* diluted in Hank's buffered sterile saline (HBSS). Infected cells were then either lysed directly (to establish total cell-associated bacteria) or treated with gentamicin (Gibco) at a concentration of 500 µg/ml for 30 min before replacement with media containing 5 µg/ml gentamycin for the remainder of the assay. Cells were then lysed in 0.2% Triton X-100 in HBSS. CFUs were enumerated by plating aliquots of lysates onto LB agar.

### Western blotting and immunoprecipitations

Cells were seeded at 1–2×10^5^ cells/well onto 24-well culture dishes 18 h prior to infection. For some experiments, PEMs were pretreated for 1 hour with the FAK- kinase inhibitor PF-228 (0.5 µM, a kind gift from J. Thomas Parsons, University of Virginia, Charlottesville, VA), rapamycin (4 µm), bafilomycin A1 (300 ng) or the Akt inhibitor AKTV/triciribine (10 µm) before infection. Cells were then rinsed twice with PBS and lysed in modified radioimmunoprecipitation assay (RIPA) buffer (50 mM Tris [pH 7.4], 1% NP-40, 150 mM NaCl, 0.5% deoxycholate, 10% glycerol, 0.1% sodium dodecyl sulfate [SDS]) supplemented with 1 mM sodium vanadate, 50 mM sodium fluoride, and a cocktail of protease inhibitors (0.1 mM phenylmethylsulfonyl fluoride and 1 µg/ml each of pepstatin, leupeptin, and antipain). Samples were loaded onto 10–16% SDS-polyacrylamide gels and probed using the indicated antibodies. For immunoprecipitations, lysates were incubated overnight with primary antibodies. The following day, immune complexes were precipitated with Protein G-Sepharose beads (Sigma), washed in RIPA buffer and loaded onto 10% SDS-polyacrylamide gels.

### Microscopy

PEMs were plated on glass coverslips (Fisher) overnight before infection with *ΔinvG* (MOI 100), *orgA::tet*, *spiA::kan* (double SPI1 and SPI2 mutant/ΔSPI1ΔSPI2) (MOI 100) or *E. coli* (MOI 100) or incubated with LPS-coated beads at a ratio of 50∶1 (bead∶cell). Beads used for phagocytosis were prepared by conjugating biotinylated LPS (InvivoGen) to 4.5 µm diameter strepavidin-polystyrene beads (Spherotech). 30 minutes after infection, media was changed to include gentamycin (500 µg/ml) to kill extracellular bacteria before replacement with media containing 5 µg/ml gentamycin for the remainder of the assay. Cells were fixed with 4% paraformaldehyde, followed by blocking and permeabilization in 10% normal goat serum (NGS) with 0.2% saponin in PBS. Primary and secondary antibodies were each diluted in blocking buffer and incubated with samples for 30 minutes–1 hour at RT. After being washed, coverslips were mounted using ProLong Gold Anti-fade (Invitrogen). Coverslips were examined with a ×100 objective on an Olympus BX51 High Magnification microscope equipped with an Olympus DP70 digitial camera. Images were acquired using ImagePro software (MediaCybernetics). For confocal microscopy, coverslips were examined using a 100× TIRF lens/ 1.49 NA objective on a Nikon C1+ Confocal scanner. Z Sections of 0.25–0.5 microns were taken and the captured 12 bit images were analyzed using Nikon Elements software. Immunostaining on tissues was performed essentially as described previously [54].

### Transmission electron microscopy

Infected PEMs were fixed in a 4% solution of paraformaldehyde containing 2.5% gluteraldehyde. Samples were incubated in 2% osmium tetroxide before dehydration in a series of ethanol washes. Samples were then embedded in epoxy resin and ultra thin sections were cut at 85 nm, picked up on 200 mesh copper grids and contrast stained with 0.25% lead citrate and 2% uranyl acetate before examination on a JEOL 1230. Images were taken at 80 kV and captured on an ultra high resolution (4K×4K) digital camera (Scientific Instruments and Applications, inc.).

### siRNA and plasmid nucleofection

20 µm siRNA oligonucleotides targeting murine Atg5 and ULK1 and non-targeting controls (Invitrogen) or 1 µg of plasmid DNA (GFP-FAK (a kind gift from A. F. Horwitz, University of Virginia, Charlottesville, VA) or HA-Akt) were nucleofected into PEMs using an Amaxa mouse macrophage nucleofector kit (Lonza) as per the manufacturer's specifications.

### Antibodies

Immunoblot analyses were performed using the following antibodies: Akt, Aktp^S473^, p70S6K, phospho-p70S6K, 4EBP1, ERK1/2, Atg5, ULK, ERK1/2, phopho-ERK1/2, p38, phospho-p38, phospho-NFκB and phospho-4EBP1 were all purchased from Cell Signaling. FAK (Santa Cruz Biotechnology, Inc.), actin (Cytoskeleton), LC3 (Novus) and FAKp^Y397^ (BD Transduction Laboratories) were purchased from the suppliers indicated. Monoclonal anti-HA (16B12) and polyclonal anti-HA (HA.11) were both purchased from Covance. For immunofluorescence studies, LC3 (Millipore), mTOR (Cell Signaling), LAMP-1 and LAMP-2 (Developmental Studies Hybridoma Bank, University of Iowa), p62 (abcam) and ubiquitin (FK2, Enzo Life Sciences) were from the suppliers indicated. Secondary antibodies included goat anti-mouse-Cy3, donkey anti-rabbit Alexa Fluor 488 and goat anti-rat Alexa Fluor 555 and were purchased from Invitrogen. For flow cytometry, TLR4 (Sa15-21; Akashi et al., 2003) was conjugated to biotin and was a kind gift from Jonathan Kagan (Harvard Medical School, Boston, MA). Anti- strepavidin-APC was purchased from Biolegend.

### Real-time PCR

*il6* (IL6), *cxcl1* (KC/CXCL1) and *tnf* (TNF-α) mRNA in confluent PEM cultures infected with *ΔinvG* (MOI 100) or untreated controls was quantified by real-time PCR. Analysis was performed using the following Applied Biosystems validated TaqMan primer-probe sets (*il6*:Mm00446190_m1; *cxcl1*:Mm04207460_m1; *tnf*:Mm00443260_g1) and the ABI PRISM SDS7000 sequence detection system (Applied Biosystems). For reverse transcription, random hexamers (1 µg) and 10 ng of total RNA were used in a final reaction volume of 20 µl containing 200 units of Superscript (Invitrogen). PCR was performed in duplicate for 40 cycles using 20% of the volume of the first strand synthesis in a total volume of 50 µl that included 25 µl of SYBR Green master mix (Applied Biosystems) and a 250 nm final concentration of primers. The Δ*CT* method was used to quantify all relative mRNA levels as described (ABI user guide, 1997), using 18 S RNA as the reference and internal standard. The TaqMan primer-probe set for 18 S RNA with the Vic/Tamra detection system was used to measure 18 S RNA in replicate samples and compared to IL6, CXCL1 and TNF-α mRNA quantifications.

### Luminol-Dependent Chemiluminescence (LDCL)

The production and measurement of ROS has been described previously with minor modifications (Cunnick 2006). Briefly 10^5^ PEMs were primed overnight with LPS (100 ng/ml). The following day, cells were washed and the media canged to phenol-free DMEM containing 10% heat-inactivated FBS in the presence of 20 µm luminol (Sigma). PEMs were stimulated with *S. typhimurium* strain *ΔινϖΓ* at an MOI of 100. LDCL was measured in a LKB-Wallac 1250 luminometer (Perkin Elmer) every 5 minutes for 1 hour at 37°C, and the counts per second (CPS) of LDCL were averaged per condition between duplicate wells.

### Infection of mice

Animals were fasted for 3–4 hours before oral-gastric infection with 1×10^9^ CFUs/mouse *Salmonella typhimurium* strain SL1344. Mice were sacrificed 5 days post-infection and the bacterial load in the ileum, liver and spleen was determined by plating serial dilutions of homogenized tissue suspensions on McConkey agar supplemented with 50 µg/ml of streptomycin.

### Cell preparation and flow cytometry

Single cell suspensions were generated from murine spleen. For flow cytometry, single cell suspensions were washed in FACS buffer (PBS containing 0.5% BSA and 1 mM EDTA) used throughout the surface staining procedure. Fc receptors were blocked by incubating with anti-CD16/32 for 15 minutes at 4°C. The following mAbs were purchased from BD Biosciences or eBioscience (unless otherwise stated), as conjugated to fluorescein isothiocyanate (FITC), PE, PerCP-cy5.5, or allophycocyanin (APC): Ly6C (AL-21), Gr-1 (RB6-8C5) and CD45 (30-F11). Isotype controls included rat IgG1 conjugated to either FITC or PE. Cells were collected on a CyAn ADP LX cytometer (Beckman Coulter) using Summit acquisition software (Dako) and analyzed using Flow Jo software (Tree Star).

### Statistical analysis

Analysis of viable CFUs recovered from the tissues of infected animals were performed using the Mann-Whitney *U* test. Student's *t* test was used for the comparison of 2 independent groups. Two-way ANOVA with Tukey's multicomponent post-test was used when comparing more than 2 independent groups. All tests were performed with Prism (GraphPad Software), and a p-value of <0.05 were considered statistically significant.

## Supporting Information

Figure S1 **FAK is not required for MyD88-dependent MAPK signaling or canonical NF-κb signaling in primary macrophages.** (A–B) WT and FAK^−/−^ PEMs were incubated with *S. typhimurium* strain *ΔinvG* for 0–60 minutes before immunoblotting with the indicated phospho-antibodies. (C) Relative *il6*, *cxcl1* and *tnf* mRNA expression in WT and FAK^−/−^ PEMs 6 hours post-infection with *S. typhimurium* strain *ΔinvG*. mRNA amounts were calculated relative to uninfected PEMs. Each point represents data from 1 set of macrophages. (D) WT and FAK^−/−^ PEMs were exposed to *ΔinvG Salmonella* opsonized in mouse serum. ROS production was measured as luminol-dependent chemiluminescence produced over 50 minutes. (E) WT and TLR4^−/−^ macrophages were incubated with *ΔinvG Salmonella* for 0–5 hours and NF-κb activity was examined by immunoblotting.
(TIF)

Figure S2 **The** ***Salmonella*** **SPI-2 system suppresses FAK and Akt signaling.** (A) Flow cytometric analysis of TLR4 expression in PEMs isolated from WT, FAK^Δmyeloid^ and TLR4^−/−^ mice. Staining of cells with secondary antibody alone is shown in the filled gray histogram. (B) WT and FAK^−/−^ PEMs were incubated with LPS-coated beads for 0–5 hours before immunoblotting for phospho-p38 (upper panel) or total p38 (lower panel). (C and E) WT and FAK^−/−^ PEMs were incubated with the non-pathogenic *E. coli* strain DH5α for 0–5 hours before immunoblotting with the indicated antibodies. In S2E, white vertical lines indicate noncontiguous lanes form a single exposure. (D and F) Levels of phosphorylated proteins were quantified by densitometry, normalized to the amount of total protein present in each sample, and expressed relative to the basal level in uninfected cells. Results are representative of 2–3 independent experiments.
(TIF)

Figure S3 **Loss of FAK does not inhibit SCV maturation.** WT and FAK^−/−^ PEMs were incubated with *S. typhimurium* strain *ΔinvG* for 1–5 hours before fixing and staining for LAMP-1. The percent of LAMP-positive SCVs is displayed to the right. N = 3.
(TIF)

Figure S4 **SPI-1-deficient** ***Salmonella*** **are inaccessible to ubiquitin or the autophagic adaptor p62.** (A) HeLa cells were infected with WT *S. typhimurium* strain SL1344 for 1 hour before analysis by IF with antibodies recognizing p62. Staining for p62 is clearly visible around bacteria validating the specificity of the α-p62 antibody (B–C) WT and FAK^−/−^ PEMs were incubated with *S. typhimurium* strain *ΔinvG* for 5 hours before analysis by IF with antibodies recognizing ubiquitin (B) or p62 (C). DAPI was used to visualize nuclei and bacteria. Bars represent 10 µm. White boxes show regions enlarged in inset panels.
(TIF)

Figure S5 **mTOR localizes with LAMP-positive SCVs in Hela cells.** Hela cells were infected with the WT *S. typhimurium* strain SL1344 for 5 hours before fixing and staining for LAMP and mTOR. DAPI was used to visualize nuclei and bacteria. White boxes show regions enlarged in lower panels.
(TIF)

Figure S6 **LC3 localizes with LAMP+ clusters of bacteria in FAK^−/−^ macrophages.** (A–B) WT and FAK^−/−^ PEMs were incubated with *S. typhimurium* strain ΔSPI1ΔSPI2 (A) or DH5α *E. coli* (B) for 5 hours before fixing and staining for LC3. DAPI was used to visualize nuclei and bacteria. In B, at least 100 bacteria were counted per condition. Values are means ± SEM, N = 3. (C) LC3 localizes with LAMP+ clusters of bacteria in FAK^−/−^ macrophages. FAK-deficient PEMs were incubated with *S. typhimurium* strain *ΔinvG* for 5 hours before fixing and staining for LC3 and LAMP-1.
(TIF)

Figure S7 **Inhibition of FAK, Akt and mTOR enhances the recruitment of LC3 to SPI-1-deficient** ***Salmonella*** **in WT PEMs.** (A–B) PEMs were pretreated with rapamycin (4 µm), Akt inhibitor AKTV/triciribine (10 µm) or left untreated (-Tx) or before infection with *S. typhimurium* strain Δ*invG* for a further 5 hours. WT macrophages were also pretreated with the FAK inhibitor PF228 (0.5 µm) for 1 hour prior to incubation with *ΔinvG Salmonella* for 5 hours. Cells were then fixed and immunostained with antibodies recognizing LC3. DAPI was used to visualize nuclei and bacteria. Bars represent 10 µm. White boxes show regions enlarged in the insets where bars represent 2 µm.
(TIF)

Figure S8 **Loss of NADPH oxidase activity does not affect the recruitment of LC3** ***to ΔinvG Salmonella*** **in macrophages.** Bone marrow-derived macrophages from gp91^phox−/−^ mice were pretreated with the FAK kinase inhibitor PF228 (0.5 µm) for 1 hour or left untreated before infection with *ΔinvG* Salmonella for 5 hours. Cells were fixed and stained for LC3. DAPI was used to visualize nuclei and bacteria. The percentage of LC3-positive Salmonella was quantified to the right. At least 100 bacteria were counted per condition. Values are means ± SEM, N = 3.
(TIF)

Figure S9 **Treatment with rapamycin, IFN-γ or Atg5 depletion did not affect the ability of** ***ΔinvG Salmonella*** **to associate with PEMs.** WT and FAK^−/−^ PEMs were either left untreated or incubated with rapamycin (4 µM), IFN-γ (50 ng/ml) overnight or depleted of Atg5 before infection with *S. typhimurium* strain *ΔinvG* for 30 minutes. Cells were then lysed directly. CFUs were enumerated by plating aliquots of lysates onto LB agar to establish total cell-associated bacteria.
(TIF)
