# Supplementary figures and images for: Activation of Focal Adhesion Kinase by Salmonella Suppresses Autophagy via an Akt/mTOR Signaling Pathway and Promotes Bacterial Survival in Macrophages
Source: PLoS Pathog. 2014 Jun 5;10(6):e1004159. doi: 10.1371/journal.ppat.1004159 (PMC4047085; doi:10.1371/journal.ppat.1004159)

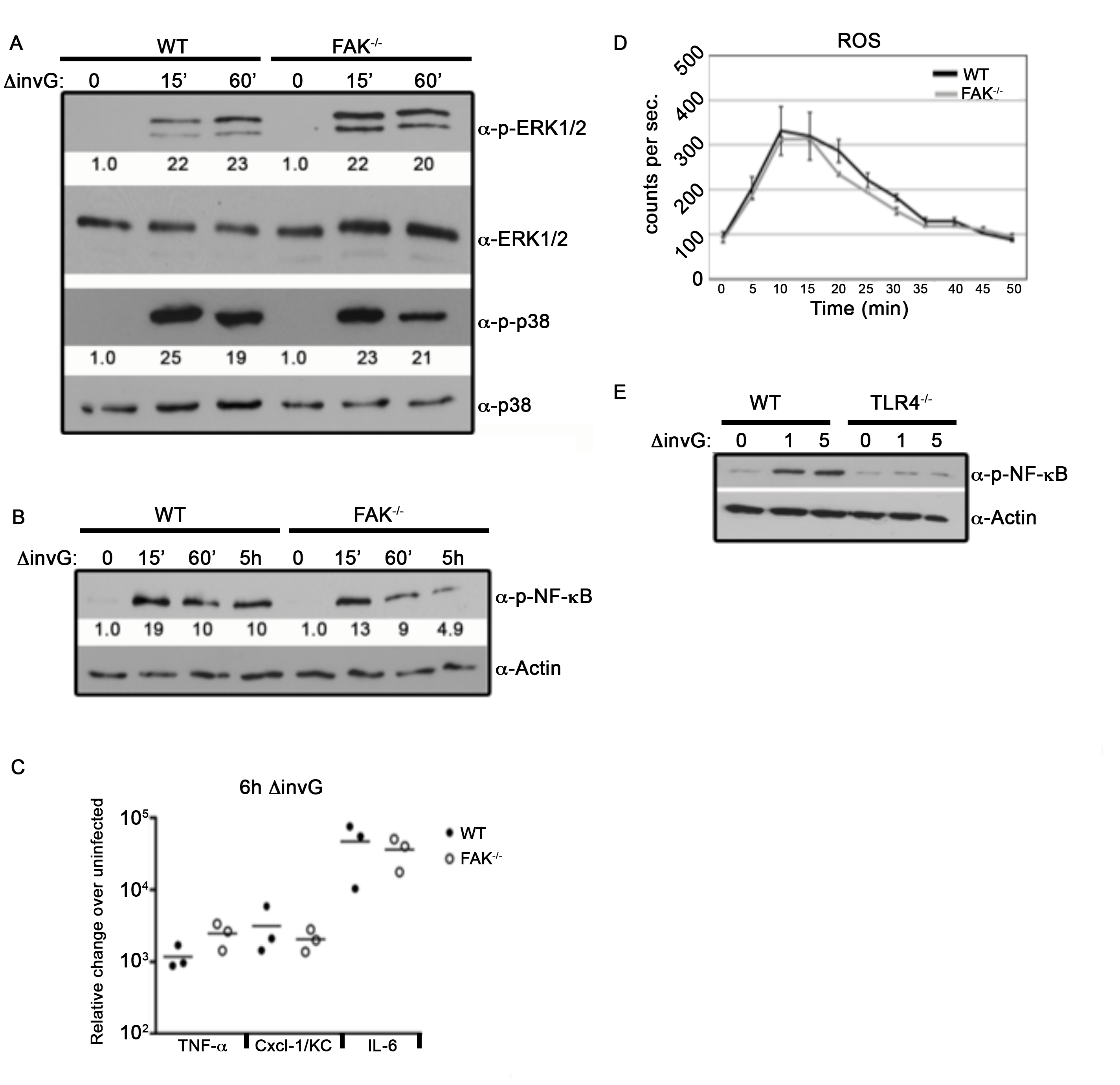

Supplement: Figure S1 — FAK is not required for MyD88-dependent MAPK signaling or canonical NF-κb signaling in primary macrophages. (A–B) WT and FAK−/− PEMs were incubated with S. typhimurium strain ΔinvG for 0–60 minutes before immunoblotting with the indicated phospho-antibodies. (C) Relative il6, cxcl1 and tnf mRNA expression in WT and FAK−/− PEMs 6 hours post-infection with S. typhimurium strain ΔinvG. mRNA amounts were calculated relative to uninfected PEMs. Each point represents data from 1 set of macrophages. (D) WT and FAK−/− PEMs were exposed to ΔinvG Salmonella opsonized in mouse serum. ROS production was measured as luminol-dependent chemiluminescence produced over 50 minutes. (E) WT and TLR4−/− macrophages were incubated with ΔinvG Salmonella for 0–5 hours and NF-κb activity was examined by immunoblotting. (TIF) [file ppat.1004159.s001.tif]

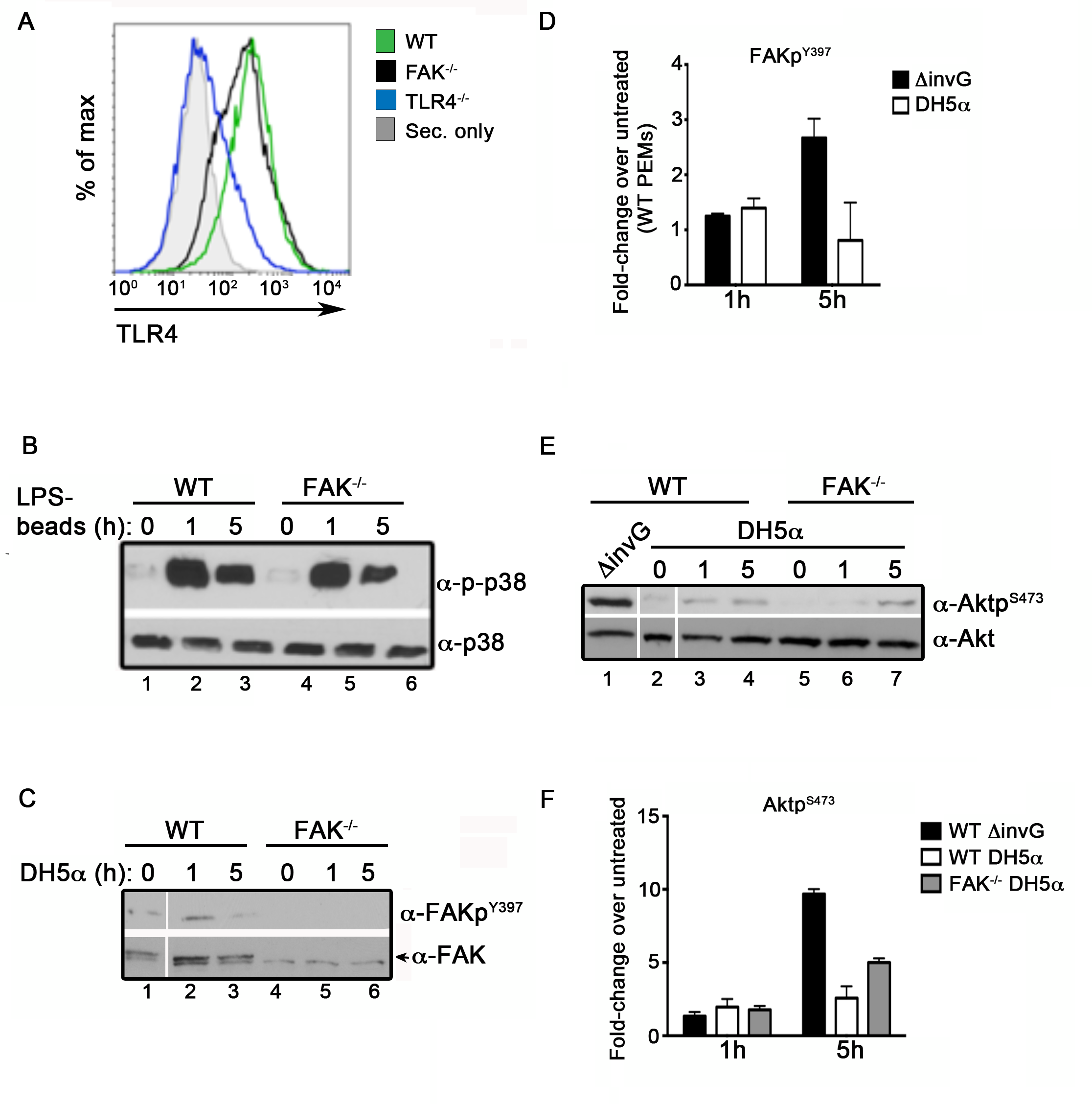

Supplement: Figure S2 — The Salmonella SPI-2 system suppresses FAK and Akt signaling. (A) Flow cytometric analysis of TLR4 expression in PEMs isolated from WT, FAKΔmyeloid and TLR4−/− mice. Staining of cells with secondary antibody alone is shown in the filled gray histogram. (B) WT and FAK−/− PEMs were incubated with LPS-coated beads for 0–5 hours before immunoblotting for phospho-p38 (upper panel) or total p38 (lower panel). (C and E) WT and FAK−/− PEMs were incubated with the non-pathogenic E. coli strain DH5α for 0–5 hours before immunoblotting with the indicated antibodies. In S2E, white vertical lines indicate noncontiguous lanes form a single exposure. (D and F) Levels of phosphorylated proteins were quantified by densitometry, normalized to the amount of total protein present in each sample, and expressed relative to the basal level in uninfected cells. Results are representative of 2–3 independent experiments. (TIF) [file ppat.1004159.s002.tif]

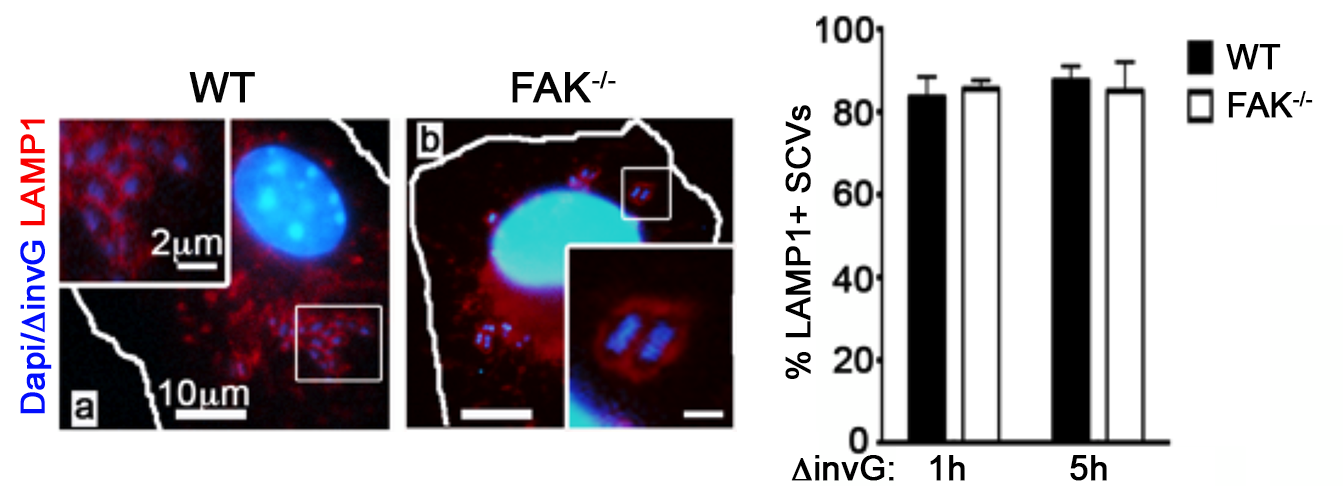

Supplement: Figure S3 — Loss of FAK does not inhibit SCV maturation. WT and FAK−/− PEMs were incubated with S. typhimurium strain ΔinvG for 1–5 hours before fixing and staining for LAMP-1. The percent of LAMP-positive SCVs is displayed to the right. N = 3. (TIF) [file ppat.1004159.s003.tif]

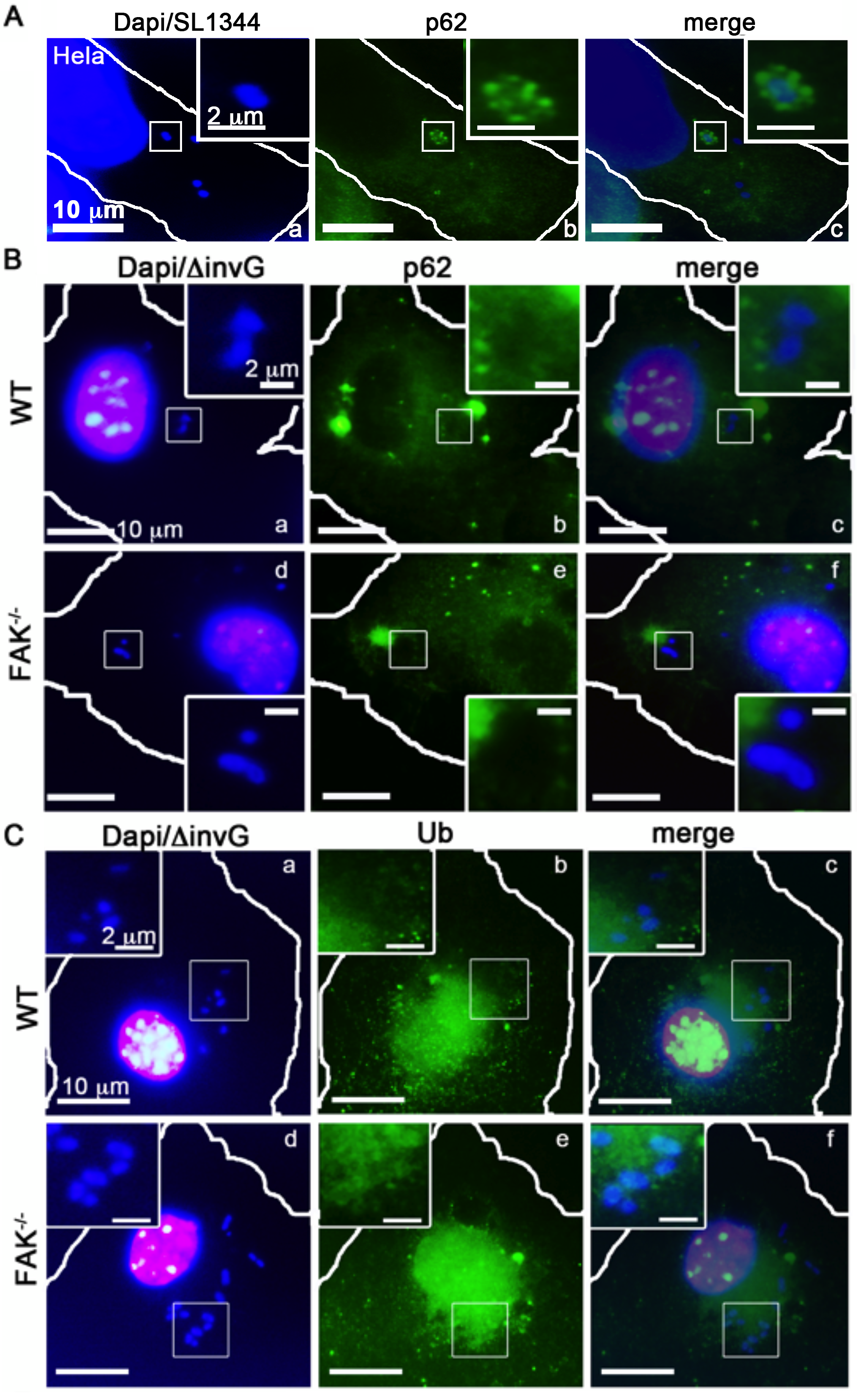

Supplement: Figure S4 — SPI-1-deficient Salmonella are inaccessible to ubiquitin or the autophagic adaptor p62. (A) HeLa cells were infected with WT S. typhimurium strain SL1344 for 1 hour before analysis by IF with antibodies recognizing p62. Staining for p62 is clearly visible around bacteria validating the specificity of the α-p62 antibody (B–C) WT and FAK−/− PEMs were incubated with S. typhimurium strain ΔinvG for 5 hours before analysis by IF with antibodies recognizing ubiquitin (B) or p62 (C). DAPI was used to visualize nuclei and bacteria. Bars represent 10 µm. White boxes show regions enlarged in inset panels. (TIF) [file ppat.1004159.s004.tif]

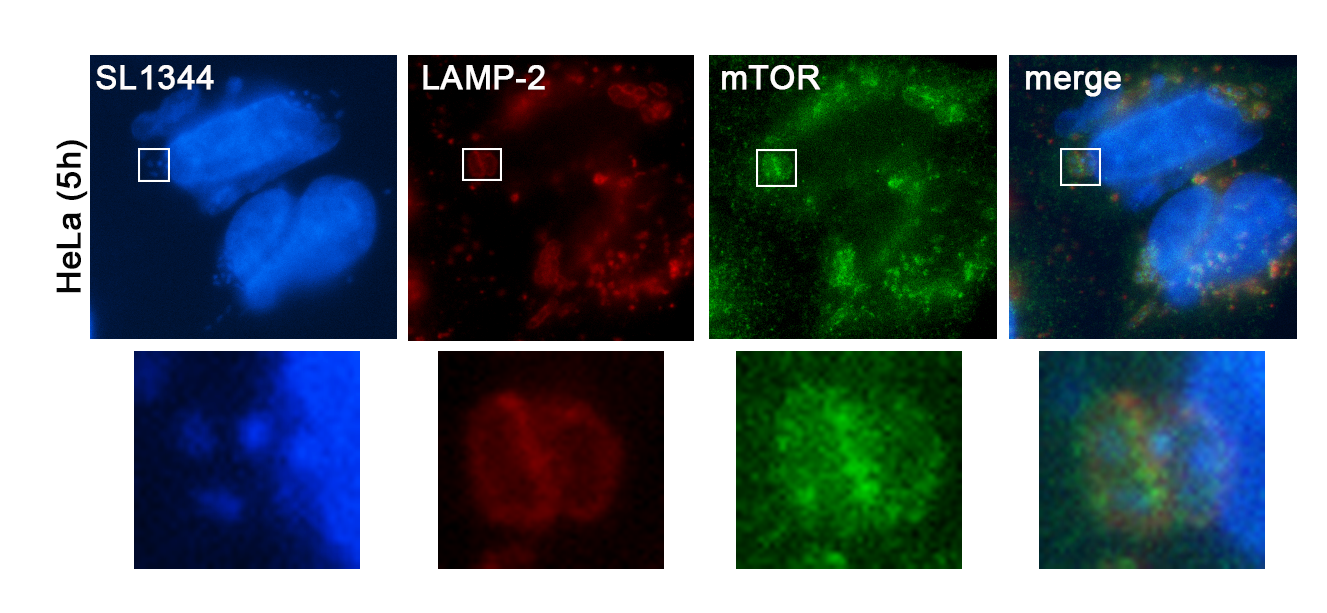

Supplement: Figure S5 — mTOR localizes with LAMP-positive SCVs in Hela cells. Hela cells were infected with the WT S. typhimurium strain SL1344 for 5 hours before fixing and staining for LAMP and mTOR. DAPI was used to visualize nuclei and bacteria. White boxes show regions enlarged in lower panels. (TIF) [file ppat.1004159.s005.tif]

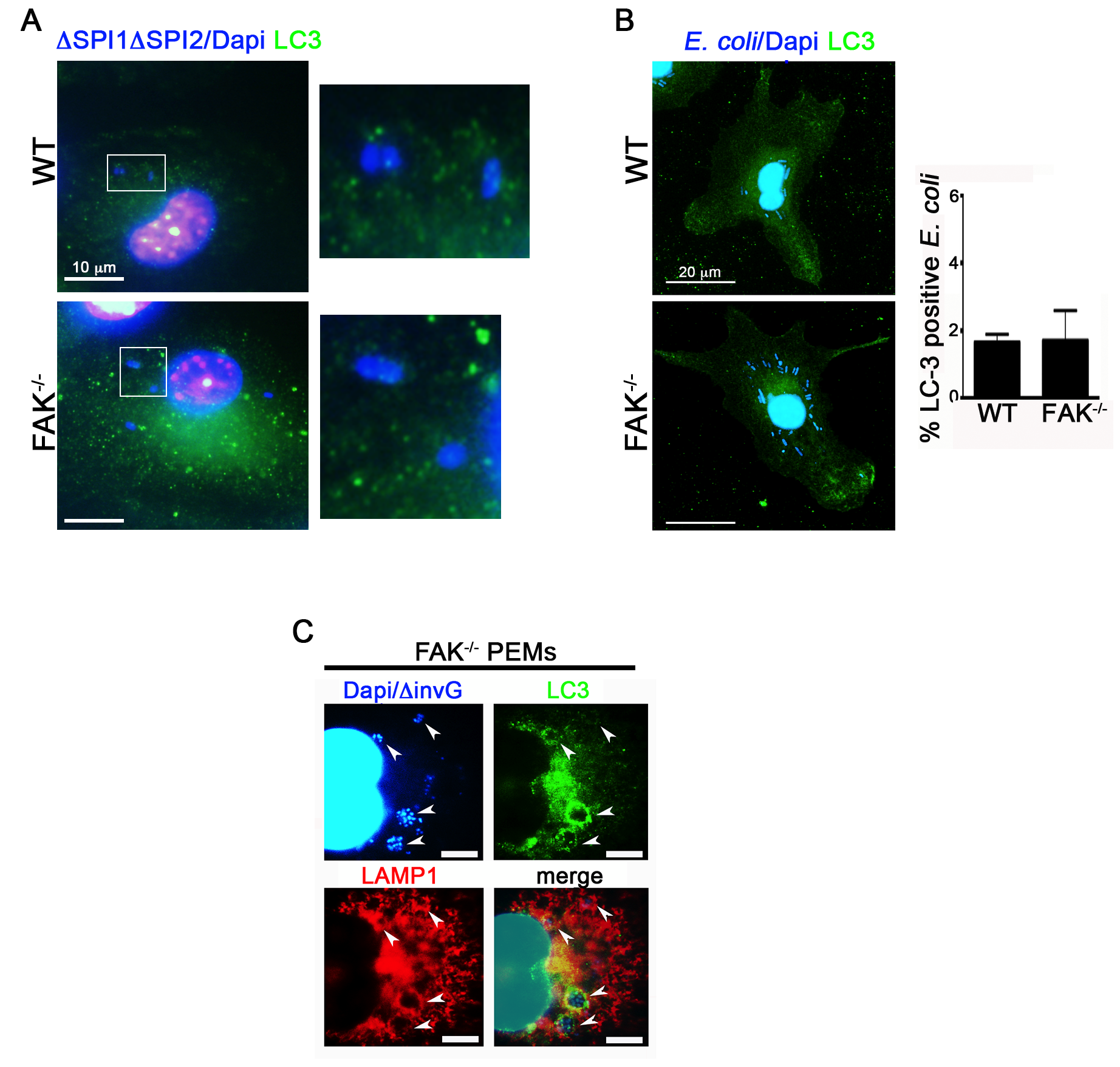

Supplement: Figure S6 — LC3 localizes with LAMP+ clusters of bacteria in FAK−/− macrophages. (A–B) WT and FAK−/− PEMs were incubated with S. typhimurium strain ΔSPI1ΔSPI2 (A) or DH5α E. coli (B) for 5 hours before fixing and staining for LC3. DAPI was used to visualize nuclei and bacteria. In B, at least 100 bacteria were counted per condition. Values are means ± SEM, N = 3. (C) LC3 localizes with LAMP+ clusters of bacteria in FAK−/− macrophages. FAK-deficient PEMs were incubated with S. typhimurium strain ΔinvG for 5 hours before fixing and staining for LC3 and LAMP-1. (TIF) [file ppat.1004159.s006.tif]

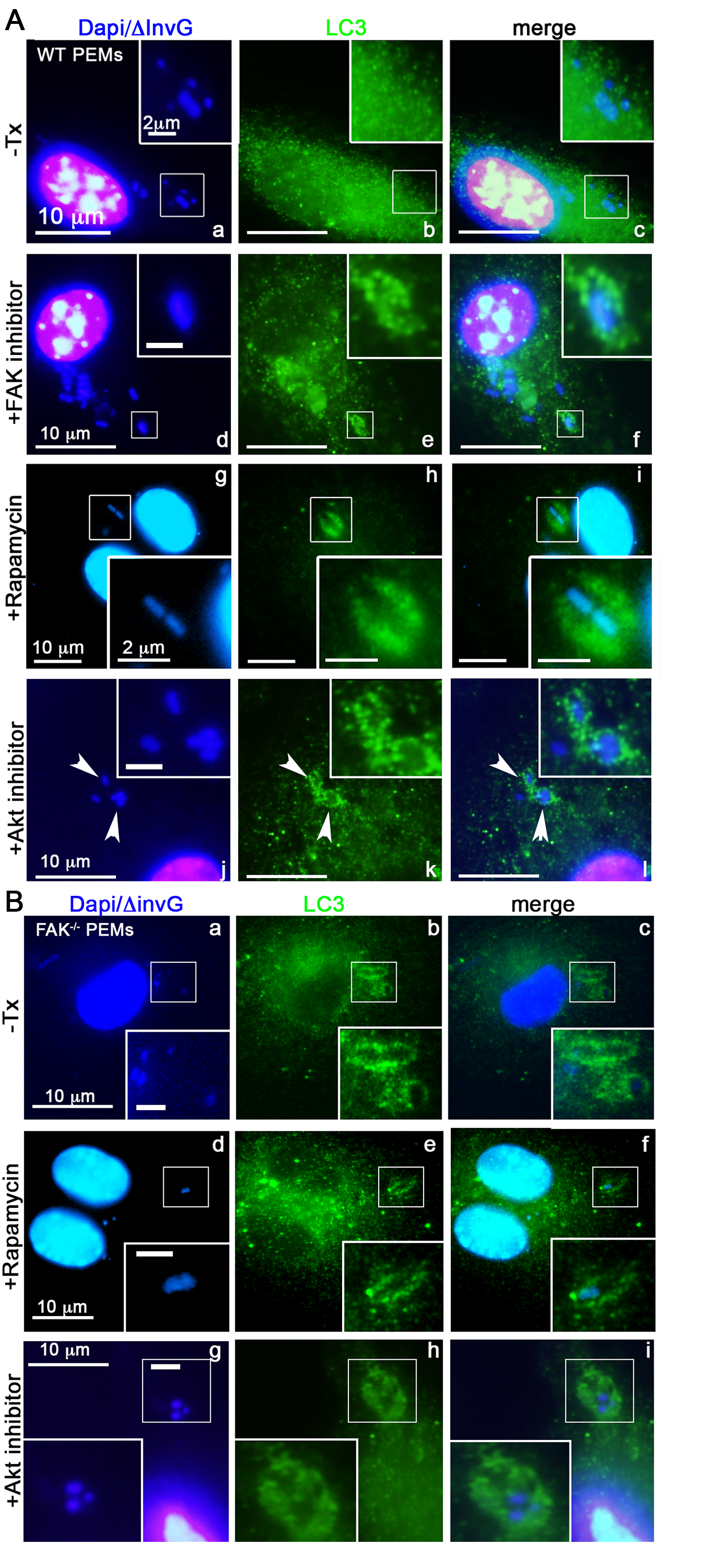

Supplement: Figure S7 — Inhibition of FAK, Akt and mTOR enhances the recruitment of LC3 to SPI-1-deficient Salmonella in WT PEMs. (A–B) PEMs were pretreated with rapamycin (4 µm), Akt inhibitor AKTV/triciribine (10 µm) or left untreated (-Tx) or before infection with S. typhimurium strain ΔinvG for a further 5 hours. WT macrophages were also pretreated with the FAK inhibitor PF228 (0.5 µm) for 1 hour prior to incubation with ΔinvG Salmonella for 5 hours. Cells were then fixed and immunostained with antibodies recognizing LC3. DAPI was used to visualize nuclei and bacteria. Bars represent 10 µm. White boxes show regions enlarged in the insets where bars represent 2 µm. (TIF) [file ppat.1004159.s007.tif]

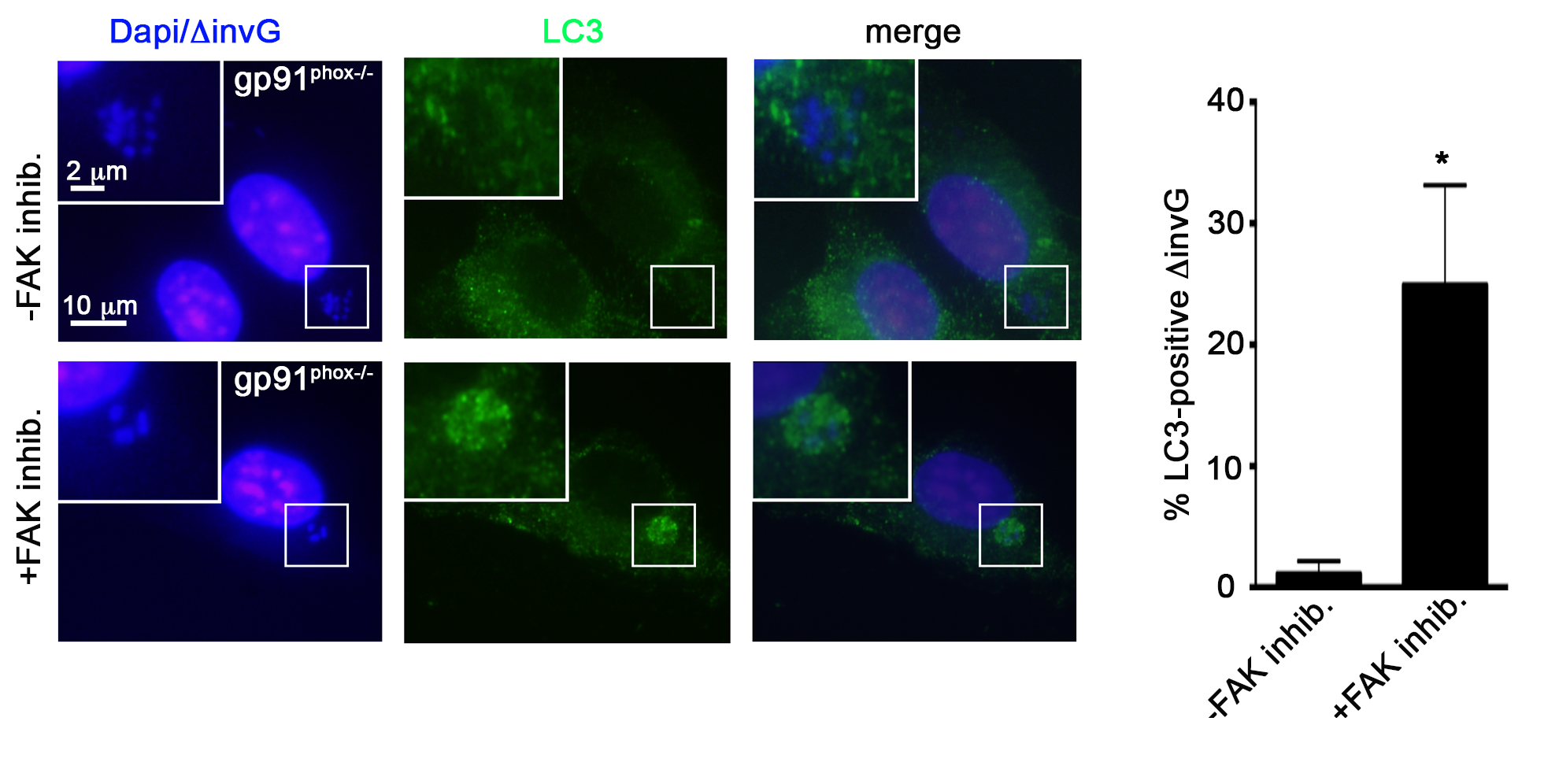

Supplement: Figure S8 — Loss of NADPH oxidase activity does not affect the recruitment of LC3 to ΔinvG Salmonella in macrophages. Bone marrow-derived macrophages from gp91phox−/− mice were pretreated with the FAK kinase inhibitor PF228 (0.5 µm) for 1 hour or left untreated before infection with ΔinvG Salmonella for 5 hours. Cells were fixed and stained for LC3. DAPI was used to visualize nuclei and bacteria. The percentage of LC3-positive Salmonella was quantified to the right. At least 100 bacteria were counted per condition. Values are means ± SEM, N = 3. (TIF) [file ppat.1004159.s008.tif]

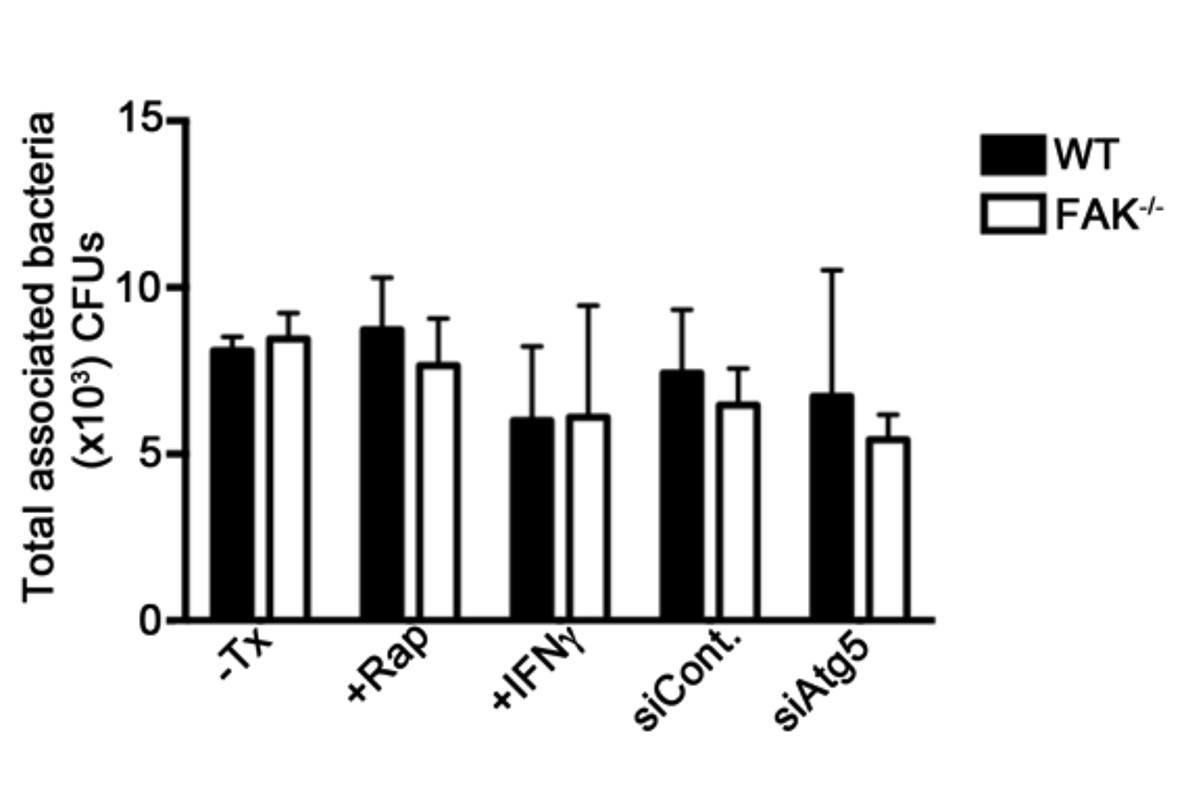

Supplement: Figure S9 — Treatment with rapamycin, IFN-γ or Atg5 depletion did not affect the ability of ΔinvG Salmonella to associate with PEMs. WT and FAK−/− PEMs were either left untreated or incubated with rapamycin (4 µM), IFN-γ (50 ng/ml) overnight or depleted of Atg5 before infection with S. typhimurium strain ΔinvG for 30 minutes. Cells were then lysed directly. CFUs were enumerated by plating aliquots of lysates onto LB agar to establish total cell-associated bacteria. (TIF) [file ppat.1004159.s009.tif]
